# A Rapid Crosstalk of Human γδ T Cells and Monocytes Drives the Acute Inflammation in Bacterial Infections

**DOI:** 10.1371/journal.ppat.1000308

**Published:** 2009-02-20

**Authors:** Matthias Eberl, Gareth W. Roberts, Simone Meuter, John D. Williams, Nicholas Topley, Bernhard Moser

**Affiliations:** 1 Department of Medical Biochemistry and Immunology, School of Medicine, Cardiff University, Cardiff, United Kingdom; 2 Institute of Nephrology, School of Medicine, Cardiff University, Cardiff, United Kingdom; Tufts University School of Medicine, United States of America

## Abstract

Vγ9/Vδ2 T cells are a minor subset of T cells in human blood and differ from other T cells by their immediate responsiveness to microbes. We previously demonstrated that the primary target for Vγ9/Vδ2 T cells is (*E*)-4-hydroxy-3-methyl-but-2-enyl pyrophosphate (HMB-PP), an essential metabolite produced by a large range of pathogens. Here we wished to study the consequence of this unique responsiveness in microbial infection. The majority of peripheral Vγ9/Vδ2 T cells shares migration properties with circulating monocytes, which explains the presence of these two distinct blood cell types in the inflammatory infiltrate at sites of infection and suggests that they synergize in anti-microbial immune responses. Our present findings demonstrate a rapid and HMB-PP-dependent crosstalk between Vγ9/Vδ2 T cells and autologous monocytes that results in the immediate production of inflammatory mediators including the cytokines interleukin (IL)-6, interferon (IFN)-γ, tumor necrosis factor (TNF)-α, and oncostatin M (OSM); the chemokines CCL2, CXCL8, and CXCL10; and TNF-related apoptosis-inducing ligand (TRAIL). Moreover, under these co-culture conditions monocytes differentiate within 18 hours into inflammatory dendritic cells (DCs) with antigen-presenting functions. Addition of further microbial stimuli (lipopolysaccharide, peptidoglycan) induces CCR7 and enables these inflammatory DCs to trigger the generation of CD4^+^ effector αβ T cells expressing IFN-γ and/or IL-17. Importantly, our *in vitro* model replicates the responsiveness to microbes of effluent cells from peritoneal dialysis (PD) patients and translates directly to episodes of acute PD-associated bacterial peritonitis, where Vγ9/Vδ2 T cell numbers and soluble inflammatory mediators are elevated in patients infected with HMB-PP-producing pathogens. Collectively, these findings suggest a direct link between invading pathogens, microbe-responsive γδ T cells, and monocytes in the inflammatory infiltrate, which plays a crucial role in the early response and the generation of microbe-specific immunity.

## Author Summary

As antibiotic resistance is spreading and posing a significant threat in many bacterial diseases, there is a need for a better understanding of host responses to infection. The precise role of an enigmatic subset of human immune cells, so-called Vγ9/Vδ2 T cells, in early infection still remains to be unveiled. These cells respond to a common molecule shared by the majority of bacterial pathogens and appear to be quickly drawn to sites of acute inflammation, where they will encounter invading microbes in the context of other immune cells, mainly granulocytes and monocytes. We here observed an unexpected interplay between microbe-activated Vγ9/Vδ2 T cells and monocytes that attracts further effector cells, enhances the activity of scavenger cells, and promotes the development of microbe-specific immunity. These findings not only improve our insight into the complex cellular interactions in early infection but may also suggest new therapies by modulating immune responses to improve host defenses and to resolve inflammatory activities.

## Introduction

The immune system has evolved to survey the body constantly for potentially hazardous structures. In order to initiate an appropriate defense, sentinel cells need to encounter ‘danger’ signals derived from invading microbes or stressed tissue [1]. Microbial signals comprise pathogen-associated molecular patterns (PAMPs) that are invariant among a broad range of organisms, allow self/non-self discrimination, and are detected by germline-encoded pattern recognition receptors [2],[3]. By contrast to this innate immune recognition, the adaptive immune response is mediated *via* somatically rearranged and clonally distributed antigen receptors on B cells and αβ T cells.

Unconventional T cells expressing γδ T cell receptors (TCRs) do not easily fit into this scheme, as they integrate features of innate and adaptive immune recognition [4]–[6]. In humans and higher primates, Vγ9/Vδ2 T cells comprise a small lymphocyte population in peripheral blood (typically 0.5–5% of all T cells [7]) that shows a striking propensity for expansion in many infections [8]. With their unique specificity for the low molecular weight compound HMB-PP [9], Vγ9/Vδ2 T cells are specialized in targeting a distinctive and vital metabolite shared by a broad range of bacteria (and some protozoan parasites) that is absent in all higher eukaryotes including humans [10],[11]. Selection and peripheral amplification of public Vγ9–Jγ1.2 clonotypes during early childhood appears to ensure rapid, innate-like responses of Vγ9/Vδ2 T cells to invading pathogens in later life [12],[13].

HMB-PP is 10'000 times more active *in vitro* than any other physiological compound [14], and the potential of microbial pathogens to stimulate Vγ9/Vδ2 T cells correlates with their ability to produce HMB-PP [15],[16]. Still, Vγ9/Vδ2 T cells may *in vivo* also respond to lower activity agonists such as isopentenyl pyrophosphate and dimethylallyl pyrophosphate released locally by necrotic host cells, and may thus alert the immune system to invading pathogens as well as to tissue damage and progressing tumors [8],[17]. Importantly, HMB-PP and isopentenyl pyrophosphate do not require presentation by human leukocyte antigen (HLA) class I and II molecules or CD1 [18], supporting a sentinel function of Vγ9/Vδ2 T cells [19],[20].

Activated Vγ9/Vδ2 T cells display distinct natural killer (NK) cell-like functions and directly eliminate infected and transformed cells, a feature that is successfully being exploited in immunotherapy trials in cancer patients [21],[22]. Another intriguing innate-like aspect is their potential to act as professional antigen-presenting cells (APCs), which includes uptake and processing of antigens and the induction of antigen-specific αβ T cell responses [23]–[25]. However, there is a paucity of data on Vγ9/Vδ2 T cells from anatomical locations other than blood and secondary lymphoid tissue, and information on the response of Vγ9/Vδ2 T cells in acute infection is especially sparse, not least due to the absence of HMB-PP-reactive γδ T cells in small animal models [4]–[6],[8]. Studies in severe combined immunodeficiency mice reconstituted with human peripheral blood mononuclear cells (PBMC) suggested that Vγ9/Vδ2 T cell may mediate rapid clearance of intraperitoneal infections, by enhancing monocyte-mediated killing of bacteria through production of interferon (IFN)-γ and tumor necrosis factor (TNF)-α [26].

Vγ9/Vδ2 T cells do not reside at common sites of pathogen entry, such as skin, lung, gastrointestinal or urinary tract, and it is unclear under which conditions they are recruited to peripheral tissues [27]. Infections trigger the local production of inflammatory chemokines, which control the composition of the cellular infiltrate [28],[29]. Importantly, a change in local chemokines is an essential factor in the transition from the neutrophil-driven immediate response to the T and B cell-driven later response to infection. The majority of circulating Vγ9/Vδ2 T cells and monocytes expresses the chemokine receptors CCR2 and CCR5 and displays similar migration characteristics [27], [30]–[32]. Vγ9/Vδ2 T cells are thus well equipped for instant relocation from the circulation and co-recruitment to inflammatory processes, which explains their accumulation at sites of infection [33].

We have here examined whether the joined extravasation of Vγ9/Vδ2 T cells and monocytes in response to pathogens is of functional relevance. Our present findings demonstrate a rapid and HMB-PP-dependent crosstalk between Vγ9/Vδ2 T cells and monocytes in the presence of microbes, leading to highly activated γδ T cells on one hand, and to monocyte differentiation into inflammatory dendritic cells (DCs) on the other hand. This interaction establishes conditions that support further recruitment of effector cells in acute infection, enhance local phagocyte activity, and create antigen-presenting cells (APCs) for the initiation of microbe-specific adaptive immunity. Importantly, these events translate directly to episodes of acute bacterial peritonitis and suggest that γδ T cells play a pivotal role in the immediate response to infection.

## Results

### Microbe-responsive γδ T cells affect monocyte morphology and survival

**Figure 1:**
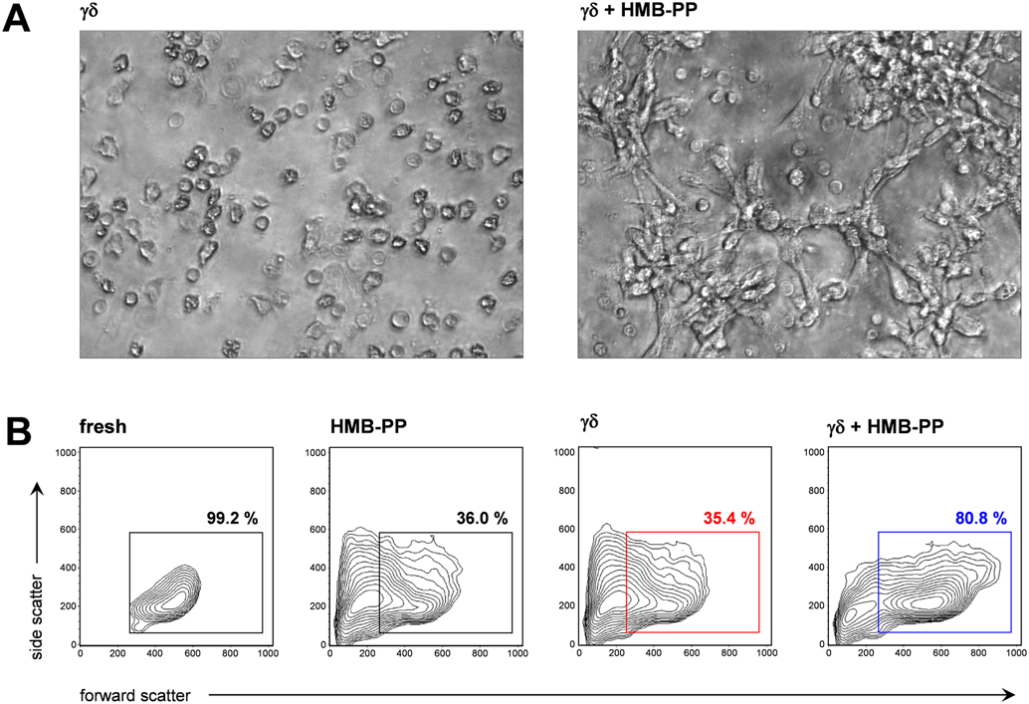
Vγ9/Vδ2 T cells promote monocyte survival and morphological changes. (*A*) Microscopic analysis of monocytes co-cultured for 18 hours with γδ T cells in the absence or presence of HMB-PP, representative of three individual donors. (*B*) Flow cytometric analysis of freshly isolated monocytes, or monocytes cultured in the presence of HMB-PP, γδ T cells, or γδ T cells+HMB-PP for 42 hours. Data shown are forward and side scatters of all CD3-negative cells, and are representative of three individual donors.

Optimum γδ T cell stimulation with the microbial metabolite HMB-PP or related compounds requires the presence of accessory cells including monocytes [18], [34]–[36]. Here, freshly isolated Vγ9/Vδ2 T cells rapidly responded to HMB-PP in the presence of autologous monocytes as judged by induction of activation markers and expression of cytokines (data not shown). However, we noticed that stimulation of Vγ9/Vδ2 T cells with HMB-PP also had a pronounced reciprocal effect on the co-cultured monocytes, and led to cluster formation and the appearance of elongated cells within 18 hours (Fig. 1A). These spindle-shaped monocytes were not observed in unstimulated monocyte-γδ T cell co-cultures, or in pure monocyte cultures incubated overnight with HMB-PP or other bacterial compounds, such as lipopolysaccharide (LPS) or peptidoglycan (PGN) (Fig. S1A). In addition, this remarkable effect was also not seen in monocytes treated overnight with granulocyte/macrophage colony-stimulating factor (GM-CSF)+IL-4, a cytokine combination giving rise to monocyte-derived DCs within a week of culture [37]; or with macrophage colony-stimulating factor (M-CSF), which induces differentiation toward macrophages [38] (Fig. S1A). In many cases, monocyte clusters formed around γδ T cells, suggesting their engagement in tight cellular interactions (Fig. S1B). In support of substantial crosstalk between these cells, the majority of monocytes survived in co-cultures with Vγ9/Vδ2 T cells in the presence of HMB-PP, whereas typically only <30% of all monocytes cultured in HMB-PP alone or together with resting Vγ9/Vδ2 T cells were still viable after two days. This ‘conditioning’ with Vγ9/Vδ2 T cells plus HMB-PP also resulted in monocyte forward/side scatter profiles that agreed with the morphologic features seen in the corresponding cultures (Fig. 1B). Collectively, these data not only confirm that monocytes provide ‘feeder’ qualities for optimum stimulation of Vγ9/Vδ2 T cells with HMB-PP but that they unexpectedly also receive reciprocal differentiation signals, which are more potent than, and distinct from, any other *in vitro* stimulus tested.

### Microbe-responsive γδ T cells induce monocyte differentiation into APCs

**Figure 2:**
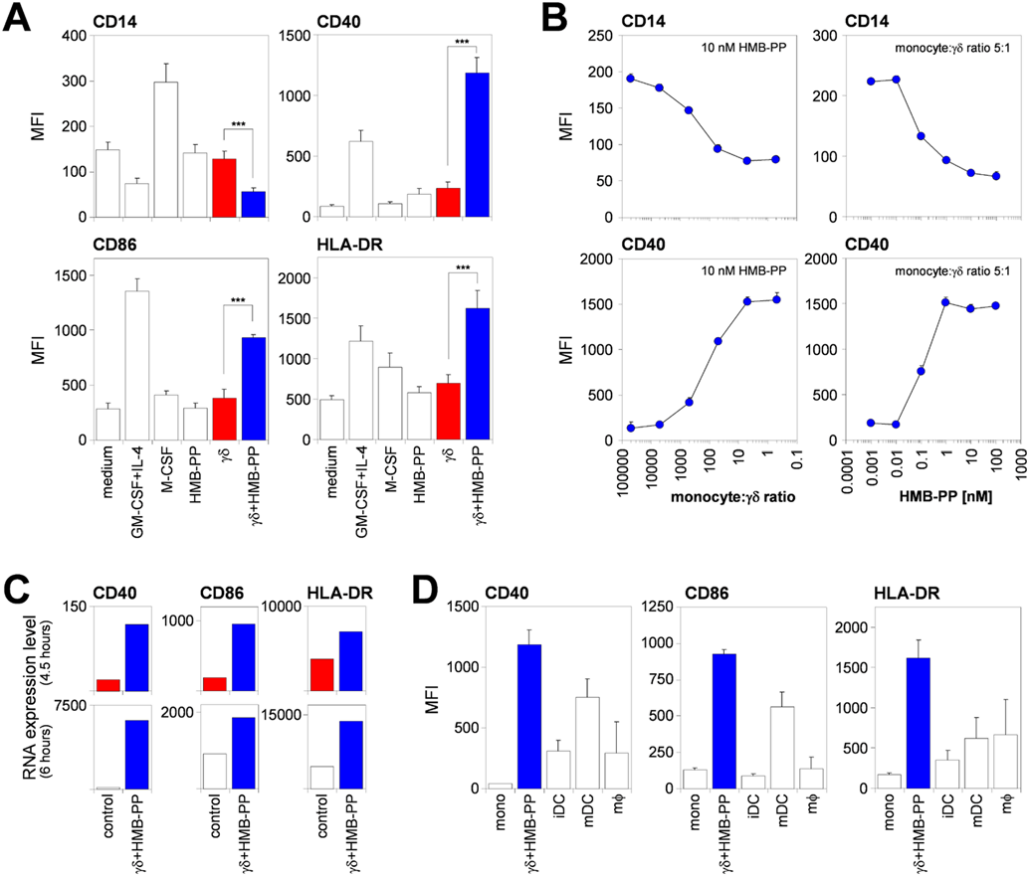
Vγ9/Vδ2 T cell-activated monocytes acquire APC markers. (*A*) Mean fluorescence intensity (MFI)±SEM of CD14, CD40, CD86, and HLA-DR for monocytes after 18 hours of culture under the conditions indicated (*n*≥4). (*B*) MFI±SD of CD14 (top) and CD40 (bottom) for monocytes co-cultured with γδ T cells at various ratios in the presence of 10 nM HMB-PP (left panels), or with γδ T cells at a ratio of 5∶1 in the presence of different concentrations of HMB-PP (right panels), as analyzed after 18 hours in triplicate. (*C*) Mean mRNA expression levels of CD40, CD86, and HLA-DRα from duplicate measurements for activated monocytes and γδ T cells sorted after 4.5–6 hours from co-cultures in the presence of HMB-PP, compared with control cells sorted from co-cultures in the absence of HMB-PP (top panels), or with freshly isolated, resting monocytes and γδ T cells (bottom panels). (*D*) MFI±SEM of CD40, CD86, and HLA-DR for freshly isolated monocytes (mono) and monocytes co-cultured with γδ T cells and HMB-PP for 18 hours, in comparison with fully differentiated immature DCs (iDC), LPS-matured DCs (mDC), and macrophages (mφ) (*n* = 3–6).

Next, we examined the phenotypic changes that occurred in monocytes during overnight co-culture with Vγ9/Vδ2 T cells and HMB-PP (abbreviated here as γδ_HMB-PP_). γδ_HMB-PP_-activated monocytes down-regulated surface CD14 within 18 hours (Fig. 2A), similarly to monocytes treated with GM-CSF+IL-4 but unlike monocytes treated with M-CSF (Table S1). At the same time, HMB-PP-stimulated Vγ9/Vδ2 T cells induced an up-regulation of the APC markers CD40, CD86, and HLA-DR on monocytes, to levels that were comparable, or even superior, to those seen with GM-CSF+IL-4 or with M-CSF. Importantly, neither resting Vγ9/Vδ2 T cells nor HMB-PP alone showed this activity. Cross-titrations confirmed a highly selective and dose-dependent effect of HMB-PP-stimulated Vγ9/Vδ2 T cells on monocytes. Down-modulation of CD14 was readily observed at an HMB-PP concentration of 0.1 nM, and at a ratio of 1 γδ T cell per 500 monocytes (Fig. 2B). Induction of APC markers in monocytes occurred with comparable efficiencies, as illustrated for CD40. Of note, induction of mRNAs for CD40, CD86, and HLA-DR in γδ_HMB-PP_-activated monocytes was very rapid and already pronounced after 4.5–6 hours (Fig. 2C). Finally, protein expression levels for APC markers on γδ_HMB-PP_-activated monocytes after only 18 hours of co-culture readily exceeded those observed on fully differentiated monocyte-derived DCs or macrophages (Fig. 2D). Collectively, these data demonstrate that γδ_HMB-PP_-activated monocytes undergo a rapid and substantial differentiation program toward an APC phenotype.

### The monocyte-γδ T cell crosstalk depends on cell-cell contact

**Figure 3:**
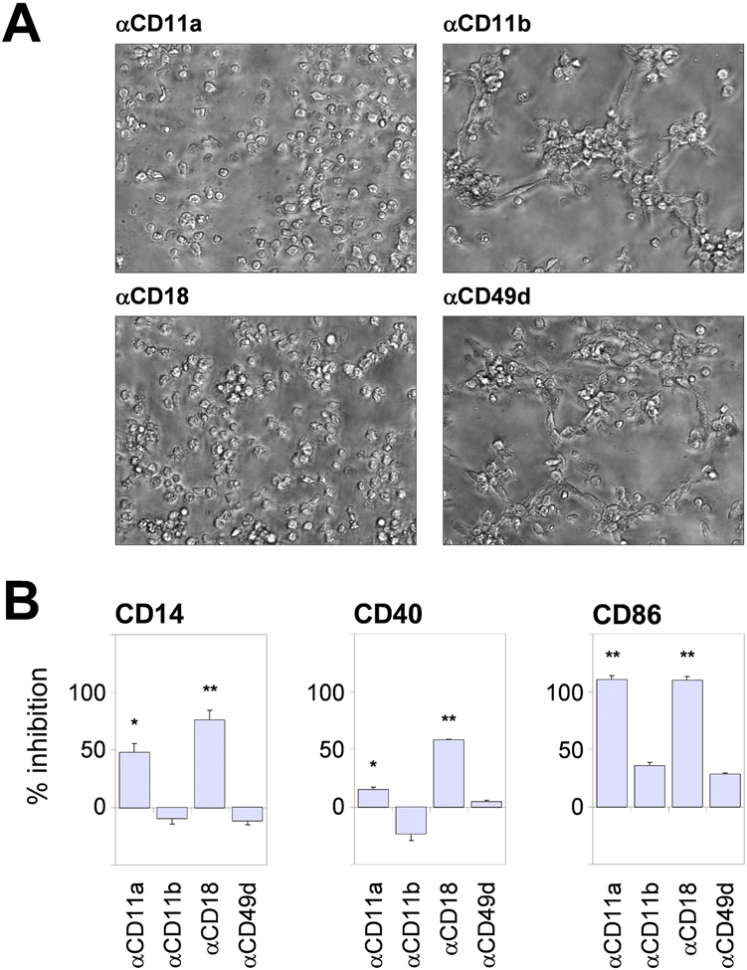
Monocyte-γδ T cell crosstalk depends on cell-cell contract. (*A*) Microscopic analysis of co-cultures of monocytes and γδ T cells incubated for 18 hours in the presence of HMB-PP and the neutralizing antibodies indicated. Data shown are representative of two individual donors. (*B*) Mean inhibitory effect±SEM of blocking antibodies on CD14 down-regulation, and CD40 and CD86 up-regulation. 0%, monocyte-γδ T cell co-cultures with HMB-PP in the absence of blocking antibodies; 100%, monocytes cultured in medium only (*n* = 4–5).

The phenotypic changes seen in γδ_HMB-PP_-activated monocytes might have stemmed from contact-dependent monocyte-γδ T cell interactions. Hence, we added neutralizing antibodies against major integrin components to co-cultures, concomitantly with HMB-PP. Blocking of CD11a or CD18 abrogated morphologic changes and cluster formation, while antibodies against CD11b or CD49d showed no such effect (Fig. 3A), indicating a crucial involvement of lymphocyte function-associated antigen-1 (LFA-1, CD11a/CD18) but not macrophage antigen-1 (Mac-1, CD11b/CD18) or very late antigen-4 (VLA-4, CD49d/CD29). As a consequence of broken cell clusters, monocytes were not transformed into APCs in the absence of LFA-1 contacts, since anti-CD11a and anti-CD18, but not anti-CD11b or anti-CD49d, antibodies inhibited CD14 down-modulation, and up-regulation of CD40 and CD86 (Fig. 3B). Collectively, these data demonstrate that the morphological changes and the rapid acquisition of APC markers by monocytes require cell-cell interactions, leading to activation of Vγ9/Vδ2 T cells in the presence of HMB-PP and reciprocal activation of monocytes.

### Microbe-responsive γδ T cells induce monocyte differentiation through cytokines

**Figure 4:**
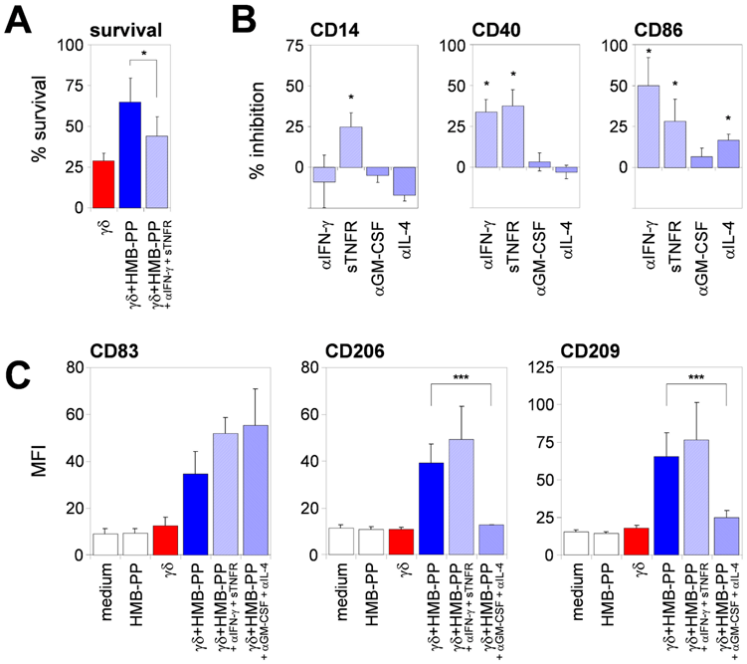
Monocyte-γδ T cell crosstalk depends on soluble mediators. (*A*) Mean percentage±SEM of surviving monocytes co-cultured with γδ T cells without or with HMB-PP, or with HMB-PP and anti-IFN-γ+sTNFR (*n* = 3), in relation to the starting numbers of monocytes in the cultures ( = 100%). (*B*) Mean inhibitory effect±SEM of the blocking reagents indicated on CD14 down-regulation, and CD40 and CD86 up-regulation. 0%, monocyte-γδ T cell co-cultures with HMB-PP in the absence of blocking antibodies; 100%, monocytes cultured in medium only (*n* = 4–5). (*C*) MFI±SEM of CD83, CD206 and CD209 for monocytes after 18 hours of culture under the conditions indicated (*n* = 3–5).

The possible contribution of soluble factors was examined in transwell experiments by measuring the response of monocytes in the lower chamber to molecules released by monocyte-γδ T cell co-cultures in the upper chamber. In this setting, HMB-PP-stimulated but not unstimulated co-cultures produced soluble factors that crossed the separating membrane and down-modulated CD14 as well as induced expression of CD40, CD86, and HLA-DR. These factors included IFN-γ and TNF-α, since addition of neutralizing antibodies against IFN-γ and soluble TNF-α receptor (sTNFR) reversed the effects on CD14 and CD40 expression in transwell cultures (Fig. S2; and data not shown). We corroborated these findings in HMB-PP-stimulated monocyte-γδ T cell co-cultures, where addition of anti-IFN-γ and sTNFR, but not anti-GM-CSF or anti-IL-4, inhibited morphologic changes and monocyte survival (Fig. 4A; Fig. S2). γδ T cell-derived IFN-γ and TNF-α also appeared to be the major regulators of cell surface CD14, CD40, and CD86 expression, whereas GM-CSF and IL-4 had only minor effects (Fig. 4B). Still, a cocktail of blocking reagents against IFN-γ, TNF-α, IL-4, and GM-CSF inhibited the γδ T cell-induced down-modulation of CD14 and up-regulation of CD40 and CD86 on monocytes more than just the combination of anti-IFN-γ+sTNFR (Fig. S3). Of note, both recombinant IFN-γ+TNF-α and GM-CSF+IL-4 promoted cell survival in pure monocyte cultures, down-modulated CD14, and induced APC marker expression.

In addition to CD40, CD86, and HLA-DR, γδ_HMB-PP_-activated monocytes expressed CD83, CD206 (mannose receptor) and CD209 (DC-SIGN) (Fig. 4C), while CD205 (DEC-205) and CD207 (langerin) were absent (Table S1). Of note, GM-CSF+IL-4 but not IFN-γ+TNF-α mimicked the effect of HMB-PP-stimulated Vγ9/Vδ2 T cells on CD206 and CD209 expression in pure monocytes (Fig. S4), and anti-GM-CSF+anti-IL-4 blocked the γδ T cell-induced expression of CD206 and CD209 in co-cultures (Fig. 4C). Neutralization of IFN-γ and TNF-α increased the levels of CD206 and CD209 even further, in line with reports showing that acquisition of ‘classical’ DC markers such as CD1a and CD209 by monocytes is dependent on IL-4 and counteracted by IFN-γ [39]. Collectively, these data demonstrate that the rapid acquisition of DC-like features by monocytes is mediated in part through the HMB-PP-driven release of IFN-γ, TNF-α, GM-CSF, and IL-4 by Vγ9/Vδ2 T cells.

### Microbe-responsive γδ T cells induce rapid expression of inflammatory mediators in monocytes

**Figure 5:**
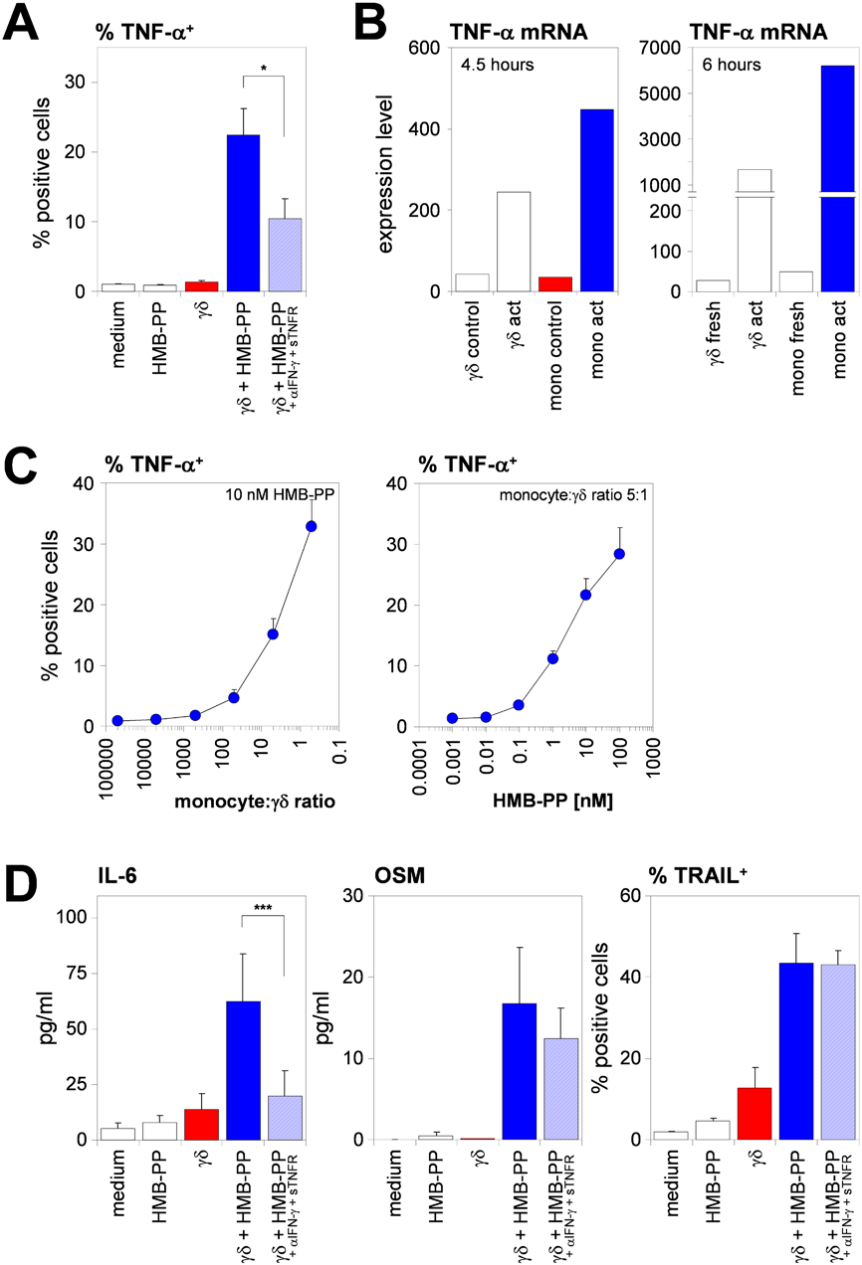
Vγ9/Vδ2 T cell-activated monocytes produce inflammatory mediators. (*A*) Mean percentages±SEM of TNF-α^+^ monocytes after 18 hours of culture under the conditions indicated (*n* = 3–7). (*B*) Mean mRNA expression levels of TNF-α from duplicate measurements for activated monocytes and γδ T cells sorted after 4.5–6 hours from co-cultures in the presence of HMB-PP, compared with control cells sorted from co-cultures in the absence of HMB-PP (left), or with freshly isolated, resting monocytes and γδ T cells (right). (*C*) Mean percentages±SD of TNF-α^+^ monocytes co-cultured with γδ T cells at various ratios in the presence of 10 nM HMB-PP (left), or with γδ T cells at a ratio of 5∶1 in the presence of different concentrations of HMB-PP (right), as analyzed after 18 hours in triplicate cultures. (*D*) Mean protein levels (pg/ml)±SEM of secreted IL-6 and OSM, and mean percentages±SEM of TRAIL^+^ monocytes after 18 hours of culture under the conditions indicated (*n* = 3–7).

Microbial sensing by DCs induces the release of a plethora of factors, the combination of which reflects the type of the encountered microorganism and the quality of the T cell response required for the control of this particular pathogen. We therefore examined the cytokine profile of γδ_HMB-PP_-activated monocytes. Addition of HMB-PP to monocyte-γδ T cell co-cultures not only induced TNF-α production in γδ T cells (data not shown), it also led to expression of significant levels of TNF-α in co-cultured monocytes (Fig. 5A). TNF-α mRNA was rapidly induced in γδ_HMB-PP_-activated monocytes and abundantly present after 4.5–6 h, at levels even exceeding those in the co-cultured γδ T cell population (Fig. 5B). Similarly as described above for CD14 and APC markers, TNF-α expression was highly dependent on the concentration of HMB-PP and the number of γδ T cells in the co-cultures (Fig. 5C). These data suggest a positive feed-back mechanism in the inflammatory response, where both monocytes and γδ T cells rapidly produce large amounts of TNF-α.

Two other inflammatory cytokines expressed by γδ_HMB-PP_-activated monocytes were IL-6 and oncostatin M (OSM), which were detectable in the supernatants of HMB-PP-stimulated co-cultures (Fig. 5D); IL-6 was confirmed to be expressed in monocytes by intracellular staining (data not shown). Expression of TNF-α and IL-6 but not OSM by γδ_HMB-PP_-activated monocytes could be blocked by anti-IFN-γ+sTNFR (Fig. 5A, 5D), and mimicked by addition of recombinant IFN-γ+TNF-α to pure monocyte cultures (data not shown). By contrast, the same monocyte-γδ T cell co-culture conditions did not lead to induction of IL-1β, IL-10, IL-12, IL-23, or IL-27, as assessed by protein and/or mRNA expression analyses (Table S1). Intriguingly, although γδ_HMB-PP_-activated monocytes expressed high levels of CD40, engagement of this receptor by soluble trimeric CD40L did not result in detectable levels of IL-12 or IL-23 but led to the release of substantial amounts of IL-6 (data not shown). Finally, neither HMB-PP-stimulation of monocyte-γδ T cell co-cultures nor treatment of monocytes with recombinant IFN-γ and/or TNF-α was able to induce significant levels of nitric oxide in monocytes (data not shown) [40]. However, these co-culture conditions led both γδ T cells (data not shown) and monocytes (Fig. 5D) to express the pro-apoptotic effector molecule TNF-related apoptosis inducing ligand (TRAIL) [41]. Collectively, these data demonstrate that crosstalk of monocytes and HMB-PP-stimulated γδ T cells leads to the rapid generation of a highly inflammatory milieu.

### Microbe-responsive γδ T cells change the migratory profile of monocytes

**Figure 6:**
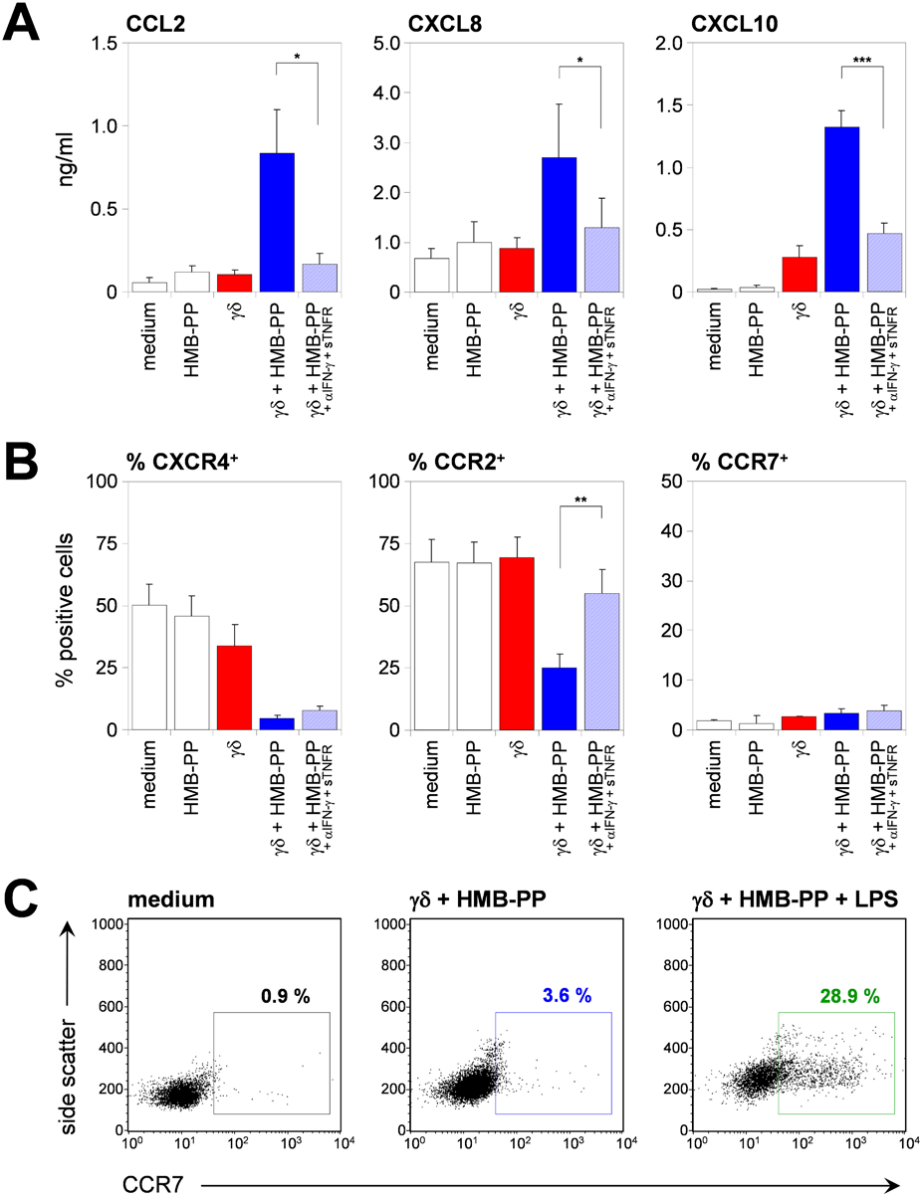
Vγ9/Vδ2 T cell-activated monocytes possess altered migratory properties. (*A*) Mean protein levels (pg/ml)±SEM of secreted CCL2, CXCL8, and CXCL10, and (*B*) mean percentages±SEM of CXCR4^+^, CCR2^+^, and CCR5^+^ monocytes after 18 hours of culture under the conditions indicated (*n* = 3–8). (*C*) Side scatter and CCR7 fluorescence for monocytes after 18 hours of culture in medium, or with γδ T cells+HMB-PP in the absence or presence of LPS; representative of two independently assessed donors.

The function of APCs is largely governed by their recruitment and relocation properties. Of note, HMB-PP-stimulated monocyte-γδ T cell co-cultures were a rich source of chemokines with known functions in acute infections, as shown for CXCL8 (IL-8) and CCL2 (MCP-1), which target neutrophils and monocytes, respectively, and CXCL10 (IP-10), one of three IFN-γ-inducible chemokines that control the recruitment of effector T cells. All three chemokines were rapidly induced in monocyte-γδ T cell co-cultures in the presence of HMB-PP and could be blocked by anti-IFN-γ+sTNFR (Fig. 6A).

Circulating monocytes express a series of chemokine receptors, including CXCR4 and the prototype monocyte receptors CCR2 and CCR5 [28]. Here, γδ_HMB-PP_-activated monocytes down-modulated surface expression of CXCR4, CCR2, and CCR5 (Fig. 6B; and data not shown), which may have resulted from chemokine-mediated receptor internalization [42]. Intriguingly, the chemokine receptor CCR7, which enables mature DCs and naïve T cells to co-localize within the T-zone of lymph nodes, was not induced under these conditions (Fig. 6B) but was readily detected when further microbial stimuli such as LPS (Fig. 6C) or PGN (not shown) were added to HMB-PP-stimulated monocyte-γδ T cell co-cultures. Collectively, these data demonstrate that γδ_HMB-PP_-activated monocytes produce cytokines and chemokines typically associated with inflammatory sites but lack factors such as CCR7 that are mobilized in response to Toll-like receptor (TLR) ligands. The chemokine profile of γδ_HMB-PP_-activated monocytes and their capacity to switch from inflammatory chemokine receptors to CCR7 is reminiscent of immature DCs undergoing maturation and acquisition of lymph node homing properties [43].

### γδ_HMB-PP_-activated monocytes present microbial antigens to αβ T cells

The resemblance of γδ_HMB-PP_-activated monocytes with inflammatory DCs prompted us to examine their ability to present antigens and induce αβ T cell activation. First, we established that monocytes co-cultured with γδ T cells retained endocytic activities, as assessed by uptake of soluble proteins, Lucifer yellow and dextran, and phagocytosis of bacteria (data not shown). These experiments ruled out the presence of inhibitory factors associated with DC maturation. Next, we studied the activation of αβ T cells in response to autologous monocytes presenting *Mycobacterium tuberculosis* purified protein derivative (PPD), which requires uptake and processing, and *Staphylococcus aureus* superantigen toxic shock syndrome toxin-1 (TSST-1), which is loaded directly onto cell surface HLA class II [23].

**Figure 7:**
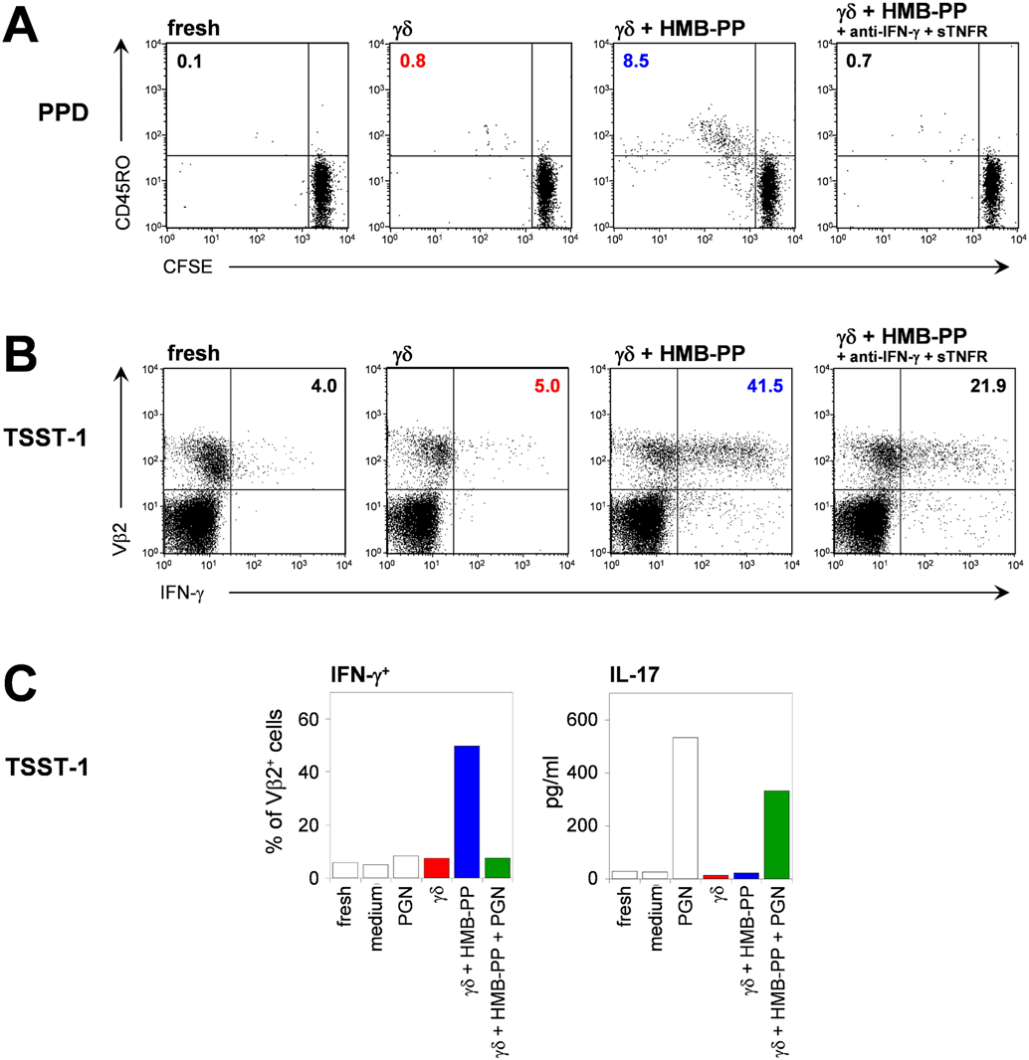
Vγ9/Vδ2 T cell-activated monocytes present bacterial antigens to autologous αβ T cells. (*A*) Freshly isolated monocytes, or activated monocytes generated for 18 hours under the conditions indicated were used as APCs and incubated with CFSE-labeled naïve αβ T cells in the presence of PPD. CFSE dilution and CD45RO expression was measured after a further 96 hours of culture. Dot plots are gated on total CD3^+^ T cells; numbers indicate the percentage of CFSE^lo^ CD45RO^+^ T cells. (*B*) Freshly isolated monocytes, or activated monocytes generated as above were loaded with TSST-1 and used as APCs for αβ T cells. Intracellular cytokine expression in responder T cells was measured after 72 hours of culture. Dot plots are gated on total CD3^+^ T cells; numbers indicate the percentage of Vβ2^+^ T cells expressing IFN-γ. No cytokine expression occurred in the absence of TSST-1. (*C*) IFN-γ and IL-17 production by naive CD4^+^ T cell cultures in the presence of TSST-1-pulsed freshly isolated monocytes or monocytes pre-treated under the conditions indicated, as detected after 72 hours by intracellular staining for IFN-γ (gated on TCR-Vβ2^+^ T cells) and ELISA for IL-17. Data in *A*–*C* are representative of two experiments performed.

γδ_HMB-PP_-activated monocytes induced a significant expansion of PPD-specific T cells from 5-(and 6-)carboxyfluorescein diacetate succinimidyl ester (CFSE)-labeled, naïve responder αβ T cells, as evidenced by the appearance of proliferating CD45RO^+^ αβ T cells after only 4 days of culture; this αβ T cell expansion was not seen in the presence of freshly isolated monocytes or monocytes co-cultured with resting Vγ9/Vδ2 T cells (Fig. 7A). Of note, neutralization of IFN-γ and TNF-α during co-culture of monocytes with HMB-PP-stimulated Vγ9/Vδ2 T cells prevented this response, which fully agrees with the observed inhibition of APC marker expression (Fig. 4). Similarly, γδ_HMB-PP_-activated monocytes served as APCs for the expansion of TSST-1-specific CD45RO^+^ Vβ2^+^ cells from CFSE-labeled, naïve responder αβ T cells (data not shown). Generation of TSST-1-specific αβ T effector cells was very robust when using γδ_HMB-PP_-activated monocytes as APCs, as evidenced by the large proportion of IFN-γ-producing Vβ2^+^ T cells, but was largely absent with control monocytes (Fig. 7B). As opposed to IFN-γ, IL-17 was not induced in TSST-1-specific responder T cells by γδ_HMB-PP_-activated monocytes, whereas γδ_HMB-PP_-activated monocytes treated with LPS or PGN were able to do so (Fig. 7B; and data not shown), confirming that TLR ligands have a direct impact on the generation of microbe-specific Th17 cells [44]. Collectively, these data suggest that monocytes turn into Th1 cell-inducing inflammatory DCs in the presence of HMB-PP-stimulated Vγ9/Vδ2 T cells and possess the capacity for further development into lymph node seeking (CCR7^+^) Th17 cell-inducing DCs upon contact with microbes *via* TLR signaling.

### Microbe-responsive γδ T cells are present in the peritoneal cavity and are potent inducers of monocyte differentiation

In order to extend our study to situations of disease, we examined peritoneal effluent cells from individuals on continuous ambulatory peritoneal dialysis (PD) (Table S2), where bacterial infection and associated inflammation remain a frequent complication. The peritoneal catheter of PD patients allows convenient, repeated, and non-invasive sampling of recruited leukocytes, and provides unique access to inflammatory scenarios *in vivo* [45].

Under stable, *i.e.* non-inflamed conditions, peritoneal effluent cells consisted mainly of CD3^+^ lymphocytes and CD14^+^ monocytes. Importantly, Vγ9/Vδ2 T cells represented a minor but detectable fraction of peritoneal leukocytes (0.06–0.28%). As these values were similar to the activation threshold in our titration experiments with peripheral monocyte-γδ T cell co-cultures (Fig. 2B), we tested whether peritoneal γδ T cells interact with peritoneal monocytes in a similar way. Indeed, addition of HMB-PP led to cluster formation and the appearance of larger, activated cells within 18 hours. This morphological change was significantly inhibited by anti-IFN-γ+sTNFR or anti-CD11a+anti-CD18 neutralizing antibodies (Fig. S5A). The Vγ9/Vδ2 T cells in these peritoneal cultures showed a dose-dependent response to HMB-PP that was already apparent at 0.1 nM, as judged by expression of CD25, CD69, and TNF-α (Fig. S5B). Moreover, HMB-PP at concentrations of >10 nM led to expansion of Vγ9/Vδ2 T cells (*i.e.*, in the absence of exogenously added cytokines) (Fig. S5B). In the same cultures, monocytes showed a corresponding dose-dependent response to HMB-PP at 0.1 nM and higher, as judged by increased forward scatter, down-modulation of CD14, up-regulation of CD40 and CD86, and induction of TRAIL and TNF-α (Fig. S5C). Collectively, these data demonstrate that Vγ9/Vδ2 T cells are present in the peritoneal cavity and able to induce rapid monocyte differentiation in the presence of minute quantities of HMB-PP.

### γδ T cell numbers and soluble inflammatory mediators are elevated in HMB-PP^+^ bacterial peritonitis

**Figure 8:**
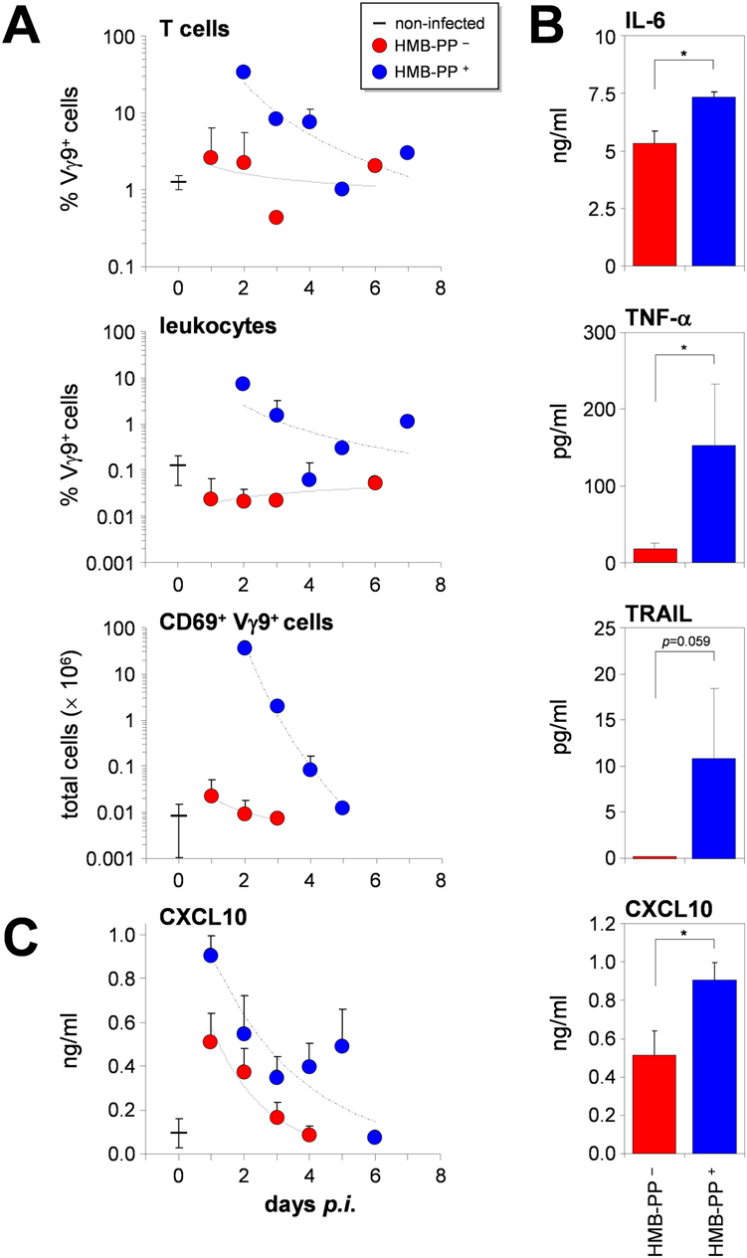
Peritoneal Vγ9/Vδ2 T cells and Vγ9/Vδ2 T cell-induced proteins are increased in HMB-PP^+^ peritonitis. (*A*) Frequencies of Vγ9^+^ T cells among CD3^+^ lymphocytes (top panel) and total leukocytes (middle panel), and absolute numbers of CD69^+^ Vγ9^+^ T cells (bottom panel), as determined in non-infected individuals (*n* = 5–7), and patients with HMB-PP^+^ (*Bacteroides splanchnicus*, *E. coli*, *Leclercia adecarboxylata*, *Proteus vulgaris*) (*n* = 5) or HMB-PP^−^ peritonitis (*Staphylococcus epidermidis*, α-hemolytic *Streptococcus spp.*) (*n* = 8–13). (*B*) Peritoneal cytokine levels±SEM on day 1 *p.i.*, and (*C*) time course of CXCL10 levels over 6 days *p.i.* in cell-free effluent fluid from patients with HMB-PP^+^ (*E. coli*, *Neisseria sp.*, *Proteus sp.*, *Pseudomonas sp.*, unspecified coliform and coryneform bacteria) (*n* = 7–10) or HMB-PP^−^ peritonitis (*Staphylococcus aureus*, *Staphylococcus epidermidis*, α-hemolytic *Streptococcus spp.*) (*n* = 13–16).

In addition to the situation in non-infected individuals, Vγ9/Vδ2 T cells were also detectable during episodes of PD-associated bacterial peritonitis. As expected from early stage infections, neutrophils represented the vast majority of peritoneal cells, while the number of Vγ9/Vδ2 T cells varied considerably (from <0.01% up to 7.3% of total leukocytes) and could amount to several million cells in the over-night effluent (Fig. 8A). Compared to stable, non-infected controls, the proportion of Vγ9/Vδ2 T cells among peritoneal CD3^+^ T cells and among total peritoneal effluent cells was considerably augmented in patients with acute peritonitis as a result of infection with the HMB-PP^+^ bacteria *E. coli*, *Leclercia*, or *Bacteroides* (Fig. 8A). This was not the case in patients infected with HMB-PP^−^ staphylococci or streptococci, which confirms *in vivo* that human Vγ9/Vδ2 T cells selectively recognize microbial pathogens capable of synthesizing HMB-PP (Table S3) [8]–[11], [14]–[16].

Peritoneal Vγ9/Vδ2 T cell frequencies and CD69 expression levels in HMB-PP^+^ peritonitis patients were consistently higher than in the peripheral blood of the same patients (data not shown), implying local recruitment/proliferation and activation in the peritoneal cavity. In addition, highest numbers of activated, CD69^+^ Vγ9/Vδ2 T cells were seen at the earliest time-points post-infection (*p.i.*) and steadily declined over the following days to the background values seen in HMB-PP^−^ bacterial infections and in non-infected individuals (Fig. 8A), underscoring an immediate as opposed to long-lasting involvement of HMB-PP-responsive γδ T cells in bacterial infections.

Finally, we examined peritoneal effluent for the presence of a series of soluble factors (IL-6, TNF-α, TRAIL, CXCL10, and OSM) that we found prominently expressed in HMB-PP-stimulated monocyte-γδ T cell co-cultures. Remarkably, all five proteins could be detected in patients samples and were higher in HMB-PP^+^ infections (*E. coli*, *Neisseria*, *Proteus*, *Pseudomonas*, unspecified coliform and coryneform bacteria) than in HMB-PP^−^ staphylococcal or streptococcal infections on day 1 *p.i.* (Fig. 8B; 9.75 and 7.38 pg/ml OSM, *n.s.*). Moreover, and in agreement with the gradual reduction in peritoneal γδ T cell numbers, cytokine levels similarly declined over the following days *p.i.* (Fig. 8C; and data not shown). Collectively, these data imply that Vγ9/Vδ2 T cells play a significant role in PD-associated acute infection caused by HMB-PP^+^ producing pathogens.

## Discussion

We have employed an *in vitro* model, composed of autologous Vγ9/Vδ2 T cells, monocytes, and the microbial metabolite HMB-PP, that mimics local conditions at early stage infections while leaving out components of the immediate (neutrophils) and adaptive (T and B cells) immune response. We hypothesized that our model would shed light on the role of Vγ9/Vδ2 T cells in the control of anti-microbial immunity. Our present findings demonstrate a reciprocal interaction between γδ T cells and monocytes within 18 hours, leading to the rapid induction of a remarkable differentiation program in monocytes. The γδ T cell-mediated effects on monocytes included enhanced survival; production of inflammatory cytokines and chemokines; and the development of DC-like characteristics, as evidenced by morphologic features, expression of APC markers, uptake and processing of antigens, and induction of antigen-specific αβ T cell responses. Of note, these inflammatory DCs were not generated when HMB-PP was omitted from the monocyte-γδ T cells co-cultures, illustrating the importance of TCR-triggering in γδ T cells for initiation of monocyte differentiation. Also, these changes in monocytes did not require the addition of exogenous cytokines and occurred within 18 hours, in contrast to what was previously reported in co-cultures with NK cells or NKT cells [46],[47]. While the mechanisms of driving monocyte differentiation toward DCs may be similar between γδ T cells and NK cells and involve the same factors, in those studies co-culture periods of up to 6 days with IL 15-activated NK cells or excess cloned NKT cells were required. In our own experiments, we observed an intimate and rapid monocyte-γδ T cell crosstalk that occurred within a few hours, at subnanomolar HMB-PP concentrations, and at ratios as low as 1 γδ T cell per monocyte. These conditions resemble early stages of infections where monocytes outnumber γδ T cells, and where low numbers of bacteria produce trace amounts of HMB-PP. Of note, we previously estimated lysates of *E. coli* [48] and *L. monocytogenes* [16] bacterial cells to contain 200–300 nM HMB-PP. Thus, we conclude that our experimental setup adequately mirrors an early aspect in acute anti-microbial defense and implies a role for inflammatory DCs, generated by short-term culture of monocytes in the presence of Vγ9/Vδ2 T cells and HMB-PP, in pathogen clearance and induction of microbe-specific T cell responses. At the same time, activated Vγ9/Vδ2 T cells may acquire APC features themselves and contribute to the transition from the innate to the adaptive phase of the immune response [23]–[25].

The pronounced effect of the microbial metabolite HMB-PP on Vγ9/Vδ2 T cells at low nanomolar concentrations is remarkable but remains elusive. Possible scenarios include direct binding of HMB-PP to the TCR, alone or in the context of a ‘presenting’ molecule on accessory cells, or the generation of a secondary Vγ9/Vδ2 T cell-specific ligand in response to HMB-PP [49]–[52]. Also, it is not clear if invading pathogens release free HMB-PP into the microenvironment, or whether HMB-PP becomes only accessible upon processing of bacteria by phagocytic cells. Studies with the intracellular pathogen *Mycobacterium tuberculosis* suggest that uptake of whole bacteria by monocytes, macrophages, or DCs is required for the recognition of HMB-PP, highlighting an essential role for monocytic cells in the activation of Vγ9/Vδ2 T cells [53]–[55]. It is conceivable that neutrophils, which are the first cells to be mobilized in response to bacterial infections, may also contribute to Vγ9/Vδ2 T cell activation. Irrespective of the underlying mechanism, the extremely potent yet highly selective activation of Vγ9/Vδ2 T cells by a single microbial compound is reminiscent of *bona fide* PAMPs and other ‘danger’ signals that our innate immune system has learnt to recognize in order to mount robust anti-microbial responses [1]–[3].

The generation of inflammatory DCs during co-culture with HMB-PP was the result of a combination of direct cell-cell contact and cytokines released by activated γδ T cells. Macroscopic changes in the co-cultures included LFA-1-dependent cell clustering as well as polarized monocyte spreading, and inhibition of integrin function prevented the formation of inflammatory DCs, in agreement with the role of LFA-1 in conjugate formation between γδ T cells and other cells [18],[56]. Transwell assays and experiments with neutralizing antibodies revealed the importance of Vγ9/Vδ2 T cell-derived soluble factors in monocyte activation, foremost IFN-γ, TNF-α, GM-CSF, and IL-4, which are co-expressed by the same Vγ9/Vδ2 T cell population upon stimulation with HMB-PP [36]. However, no cytokine combination fully substituted for HMB-PP-stimulated Vγ9/Vδ2 T cells, and complete inhibition was not achieved with a cocktail of blocking reagents against IFN-γ, TNF-α, GM-CSF, and IL-4, implying additional soluble and/or cell-associated components in promoting monocyte activation, including IL-13, CD40L, and integrin ligands [31],[36]. Thus, we conclude that HMB-PP-stimulated Vγ9/Vδ2 T cells are perfectly equipped to interact with monocytes by providing a whole range of factors necessary for monocyte survival and differentiation into inflammatory DCs. Clearly, these findings differ from the reported effect of γδ T cell-derived IFN-γ and TNF-α on monocyte-mediated killing of bacteria [26] or on the maturation of immature DCs [55], [57]–[59].

The overall outcome of our *in vitro* co-cultures was a milieu rich in inflammatory mediators that gave rise to cells with characteristics of immature DCs. We did not detect IL-1β, IL-12, IL-23, or IL-27 in the culture supernatants, a fact that may be explained by the absence of TLR ligands or other ‘danger’ signals. Despite uniform expression of CD40 on γδ_HMB-PP_-activated monocytes, signaling through this receptor by means of co-culture with CD40L^+^ Vγ9/Vδ2 T cells or activation with soluble CD40L did not lead to substantial levels of IL-12 or IL-23, further documenting the paramount importance of synergism with microbial products [60]. The inflammasome-controlled processing of IL-1β is induced by numerous microbial ligands [61], and the absence of IL-1β (and IL-23) in our co-cultures could explain the observed inability of inflammatory DCs to induce the differentiation of naïve αβ T cells into Th17 cells [44]. In agreement, we were able to ‘correct’ this deficit by adding LPS or PGN to our co-cultures, which resulted in inflammatory DCs capable of inducing Th17 cells. Finally, the lymph node homing receptor CCR7 was completely absent in inflammatory DCs but became expressed upon treatment with LPS or PGN. We therefore conclude that our co-culture system allows a view at the cellular cross-talk between two types of simultaneously recruited immune cells that occurs in the absence of TLR signaling. This model will be useful for studying the parameters that determine the ‘quality’ of microbe-specific αβ T cell responses (Th1, Th2, Th17, Tfh, Treg) by treating inflammatory DCs with defined microbial compounds or whole pathogens.

It is conceivable that our model of γδ_HMB-PP_-induced inflammatory DCs is more relevant to induction of adaptive immunity in response to acute infections as opposed to monocyte-derived DCs that require one week of *in vitro* culture for development [37]. Recent studies in mice provided compelling evidence that circulating monocytes are able to develop into DCs *in vivo* [62],[63]. Murine Gr-1^+^ CCR2^+^ monocytes (which correspond to the CD14^high^ human blood monocytes used in the present study) are recruited to sites of infection where they differentiate into inflammatory DCs [64],[65]. The factors responsible for the conversion of blood monocytes into DCs in humans and the kinetics by which they act are largely unknown, where access to tissue material at early stage infections is limited. Evidently, inflammatory chemokines, *e.g.* those produced under the settings of bacterial invasion, will selectively recruit blood monocytes expressing the corresponding chemokine receptors while factors provided by local tissue-resident cells and microbes will affect survival and differentiation of recruited monocytes.

The powerful monocyte-γδ T cell crosstalk reported here translates directly to episodes of bacterial peritonitis. Our data demonstrate that Vγ9/Vδ2 T cells in peritonitis patients infected with HMB-PP^+^ bacterial species were always highest at the earliest time points when infected peritoneal samples could be retrieved (day 1–2 *p.i.*), and then gradually declined over the next few days. In contrast, Vγ9/Vδ2 T cell frequencies and total numbers in patients infected with HMB-PP^−^ bacterial species did not vary from the stable, non-inflamed situation, and remained constant over the course of one week after the onset of infection. Most remarkably, this initial peak and rapid resolution of Vγ9/Vδ2 T cell numbers in HMB-PP^+^ peritonitis clearly preceded the delayed influx of αβ T cells (day 3–4 *p.i.*) yet overlapped with the early wave of neutrophils (day 1–2 *p.i.*) that was observed previously in PD-associated peritonitis patients [45], [66]–[68]. An attractive hypothesis would predict that some of the mediators we identified in our *in vitro* model control neutrophil recruitment and function (IFN-γ, IL-6, OSM) [66],[67] as well as neutrophil turnover (TRAIL) [69]. Accordingly, we could demonstrate that responses to HMB-PP of unfractionated peritoneal cells were indistinguishable from those seen in our *in vitro* model, as evidenced by cytokine production (including IFN-γ, TNF-α, and TRAIL) and induction of APC markers on monocytes. Thus, Vγ9/Vδ2 T cells are ideally positioned to contribute to immediate infection control and to support microbe-specific adaptive immune responses in patients with HMB-PP^+^ bacterial infections. Ongoing studies are designed to examine the monocytic infiltrate and DC subsets in acute bacterial peritonitis and detect phenotypic and functional differences between HMB-PP^+^ and HMB-PP^−^ infections. Of note, disproportionate monocyte-γδ T cell crosstalk may result in excessive production of inflammatory mediators, possibly explaining why episodes of HMB-PP^+^ peritonitis are associated with a 2–3fold increased risk of PD technique failure (removal of the peritoneal catheter, transfer to hemodialysis, and/or patient death) and implying a role for γδ T cells in the nature and severity of the inflammatory response to pathogens (our unpublished observations).

In summary, our *in vitr*o and *ex vivo* data support a model where Vγ9/Vδ2 T cells bridge innate and adaptive immune mechanisms in response to infection with HMB-PP-producing pathogens. At the earliest stage of infection, microbial products activate local macrophages and tissue cells to produce neutrophil-specific (CXCL8 and related chemokines) and monocyte/γδ T cell-specific chemokines (CCL2-5) [70]. Freshly recruited Vγ9/Vδ2 T cells interact with monocytes and become activated by microbial-derived HMB-PP, which in turn leads to substantial cytokine secretion and the generation of inflammatory DCs. Following antigen-uptake and processing, newly generated DCs upregulate CCR7 in response to microbes, and relocate to the draining lymph nodes where they instruct microbe-specific effector T cells. Thus, Vγ9/Vδ2 T cells set the stage for early adaptive immune responses while invading microbes determine the choice of play.

## Materials and Methods

### Ethics statement

This study was conducted according to the principles expressed in the Declaration of Helsinki and under local ethical guidelines (Bro Taf Health Authority, Wales). The study was approved by the South East Wales Local Ethics Committee under reference number 04WSE04/27. All patients provided written informed consent for the collection of samples and subsequent analysis.

### Patients

43 patients on PD for ≤5 years were recruited from the Peritoneal Dialysis Unit, Cardiff University School of Medicine (Table S2). Diagnosis of acute peritonitis was based on the presence of abdominal pain, a cloudy peritoneal effluent with >10^5^ leukocytes per ml, and a positive microbiological culture. The day of the first appearance of leukocytes in the effluent was defined as day 1 post-infection. The causative organisms of bacterial peritonitis were divided into HMB-PP^−^ (*Enterococcus*, *Staphylococcus*, *Streptococcus*) and HMB-PP^+^ species (*Bacteroides*, *Corynebacterium*, *Escherichia*, *Leclercia*, *Neisseria*, *Proteus*, *Pseudomonas*, and other coliform or coryneform bacteria), in accordance with the distribution of the non-mevalonate pathway of isoprenoid biosynthesis across their genomes (Table S3) [8],[11]. Patients with bacterial episodes of peritonitis were uniformly treated with a standard regime of ciprofloxacin and vancomycin according to the guidelines of the International Society for Peritoneal Dialysis (ISPD).

### Cell isolation

Peritoneal cells were harvested from chilled overnight dwell effluents [45]; cell-free supernatants were stored at −70°C. PBMC were isolated from peripheral blood using Lymphoprep (Axis-Shield). Monocytes (>99%) were purified from PBMC of healthy volunteers using anti-CD14 microbeads (Miltenyi). Vγ9/Vδ2 T cells (99.04±0.65% Vγ9^+^, mean±SD) were purified from CD14-depleted PBMC using monoclonal antibodies (mAbs) against Vγ9-PE-Cy5 (Immu360; Beckman-Coulter) and anti-PE microbeads (Miltenyi). Untouched bulk αβ T cells (>95%) and naïve CD4^+^ T cells (>95%) were purified from γδ T cell-depleted PBMC using the pan-T cell isolation kit II and the naïve CD4^+^ T cell isolation kit (Miltenyi), respectively. Immature DCs were derived from monocytes over 6 days in 50 ng/ml GM-CSF and 10 ng/ml IL-4 (Peprotech); mature DCs were obtained from DCs by adding 100 ng/ml LPS (Sigma) for 15 h. Macrophages were derived from monocytes over 6 days in 50 ng/ml M-CSF (Peprotech).

### Flow cytometry

Cells were analyzed on a four-color FACSCalibur supported with CellQuest (BD Biosciences), using mAbs against pan-TCRγδ (11F2), TCR-Vδ2 (B6.1), CD3 (SK7, UCHT1, HIT3a), CD4 (RPA-T4), CD14 (MOP9), CD25 (M-A251), CD45RA (HI100), CD45RO (UCHL-1), CD69 (FN50), CD83 (HB15e), CD86 (2331), HLA-DR (L243), CCR5 (2D7), and CXCR4 (12G5) (all from BD Biosciences); TCR-Vβ2 (MPB2D5), TCR-Vγ9 (Immu360), CD40 (mAB89), CD206 (3.29B1.10), and CD207 (DCGM4) from Beckman Coulter; CD209 (120507) and CCR2 (48607.211) from R&D Systems; CD205 (DEC-205) from eBioscience; TCR-Vδ1 (TS8.2) from Endogen; and rat anti-CCR7 (3D12) from Dr. M. Lipp (Max Delbrück Center for Molecular Medicine, Berlin, Germany); together with appropriate isotype controls and secondary reagents. For detection of intracellular cytokines, brefeldin A (Sigma) was added to cultures at 10 µg/ml 4 hours prior to harvesting. Surface-stained cells were labeled using the Fix&Perm kit (eBioscience) and mAbs against IFN-γ (45.15), TRAIL (RIK-2) (BD Biosciences), TNF-α (188) (Beckman Coulter), IL-6 (AS12), IL-10 (JES3-9D7), and IL-17 (64DEC17) (eBioscience). Monocytes in co-cultures were identified based on their appearance in forward/sideward scatter, lack of CD3 expression and residual expression of CD14; γδ T cells were gated on CD3^+^ Vγ9^+^ lymphocytes.

### Monocyte cultures

The medium used was RPMI-1640 with 2 mM L-glutamine, 1% non-essential amino acids, 1% sodium pyruvate, 50 µg/ml penicillin/streptomycin, 50 µM β-mercaptoethanol, and 10% fetal calf serum (Invitrogen). Monocytes were co-cultured with γδ T cells at a ratio of 5–10 monocytes per γδ T cell in the presence of 10 nM synthetic, *i.e.* LPS-free HMB-PP [14], with no additional stimuli. Monocytes incubated with γδ T cells or HMB-PP alone served as controls. In transwell experiments, monocytes were separated from monocyte-γδ T cell co-cultures by 0.4 µm pore polycarbonate membranes (Fisher Scientific). Alternatively, monocytes were cultured with 10 ng/ml IFN-γ, 20 ng/ml TNF-α, 50 ng/ml GM-CSF, 50 ng/ml M-CSF, 10 ng/ml IL-1β, 10 ng/ml IL-4, or 50 ng/ml IL-6 (Peprotech); 100 ng/ml trimeric CD40L with 1 µg/ml enhancer (Alexis); 100 ng/ml LPS from *Salmonella abortus equii* (Sigma); 5 µg/ml PGN from *Staphylococcus aureus* (Sigma); or combinations of which. Blocking reagents used were anti-IFN-γ (25718), anti-GM-CSF (3209), anti-IL-4 (3007), and anti-CD40L (40804) from R&D Systems; anti-CD11a (TS1/22), anti-CD11b (OKM1), and anti-CD18 (TS1/18) from Dr. R. Pardi (DIBIT-Scientific Institute San Raffaele, Milano, Italy); anti-CD49d (HP2/1, HP2/11) from Dr. F. Sánchez-Madrid (Hospital Universitario de la Princesa, Madrid, Spain); and sTNFR p75-IgG1 fusion protein (etanercept, Enbrel®) from Amgen; alone or in combination at 10 µg/ml each.

### APC assays

Monocytes were activated as described above except that the co-cultured γδ T cells were irradiated at 12–19 Gray. Freshly isolated or pre-activated monocytes were washed and used as APCs for autologous bulk TCRαβ^+^ T cells or naïve CD4^+^ T cells, at a ratio of 5–10 αβ T cells per monocyte; for proliferation assays, responder αβ T cells were pre-labeled with CFSE (Molecular Probes). TSST-1 (Toxin Technology) was pulsed directly onto APCs at 1 ng/ml for 1 h; PPD (Statens Serum Institut) was added to the T cell cultures at 1 µg/ml.

### Microscopic analysis

Monocytes were co-cultured with γδ T cells as described above except that in some assays γδ T cells were purified using biotinylated pan-TCRγδ mAbs (11F2; BD Biosciences) and anti-biotin microbeads (Miltenyi), and labeled with PKH26 (Sigma). Photographs were taken from live cultures at a magnification of 200×, using a Leica DM IRBE inverted microscope with a Hamamatsu ORCA-ER camera supported with OpenLab 3.1.7 (Improvision). Images were processed with Photoshop 6.0 (Adobe).

### Real-time PCR

γδ T cells and monocytes from 4.5–6 hours co-cultures with or without HMB-PP were sorted to >99.5% purity each on a MoFlo machine (Cytomation), using mAbs against Vγ9, Vδ2, CD3, CD4, and CD14. Freshly isolated γδ T cells and monocytes served as controls. Total RNA was isolated using Trizol (Invitrogen) and reverse transcribed with SuperScript II in the presence of 500 µg/ml random hexamer primers and 100 mM dNTPs (Invitrogen). Real-time PCRs were run on ABI Prism 7000 and 7900HT systems, using 2×ABI master mix (Applied Biosystems), 0.9 µM forward and reverse primers, and 0.25 µM 5′-FAM and 3′-BHQ1 labeled probes (Microsynth). Primer sequences are listed in Table S4; amplification efficiencies were between 1.92 and 2.0 (*R* ^2^>0.95). Relative gene quantification was performed in duplicate using the 2^−ΔΔ*C*^_T_ method. Results were expressed as expression levels relative to 1,000 copies of cyclophilin A.

### Culture supernatants and effluent samples

Cell-free peritoneal effluents and culture supernatants were analyzed using ELISA kits for IL-1β, IL-6, IL-12p70, IL-17, IL-27, CXCL10, TRAIL, and OSM (R&D Systems); IFN-γ, TNF-α, CXCL8, and CCL2 (BD Biosciences); and IL-23 (eBioscience). Monocyte-derived nitric oxide was assessed using the Total Nitric Oxide and Nitrate/Nitrite Parameter Assay Kit (R&D Systems). All samples were measured in duplicate on a Dynex MRX II reader.

### Statistical analysis

Data were analyzed using two-tailed Student's *t*-tests (GraphPad Prism 4.0), with differences considered significant as indicated in the figures: *, *p*<0.05; **, *p*<0.01; ***, *p*<0.001.

## Supporting Information

Table S1 Schematic summary of the phenotypical characterization of freshly isolated monocytes, monocytes cultured in medium or with LPS, recombinant GM-CSF+IL 4, M-CSF, or IFN-γ+TNF-α, and monocytes cultured in the presence of γδ T cells+HMB-PP, based on up to 17 independently assessed donors. Expression levels of the markers indicated were assessed after 18 hours of stimulation: −, absent or marginal; +, present; ++, strongly expressed; +++, very strongly expressed; *n.d.*, not determined.
(0.02 MB PDF)

Table S2 Peritoneal dialysis patients with and without acute bacterial peritonitis that were analyzed in this study. *n.a.*, not applicable.
(0.02 MB PDF)

Table S3 Distribution across bacterial genomes of coding sequences for 3-hydroxy-3-methylglutaryl-CoA (HMG-CoA) reductase (*hmgr*), the key enzyme of the classical mevalonate pathway; and for HMB-PP synthase (*ispG*) and HMB-PP reductase (*ispH*), two enzymes of the alternative non-mevalonate pathway of isoprenoid synthesis. Genomic and protein sequences from bacterial species of relevance for the present study were retrieved from the public servers at the National Center for Biotechnology Information (http://www.ncbi.nlm.nih.gov), Wellcome Trust Sanger Centre (http://www.sanger.ac.uk), Washington University Genome Sequencing Center (http://genome.wustl.edu), and Baylor College of Medicine Human Genome Sequencing Center (http://www.hgsc.bcm.tmc.edu). Sequence homologies were analyzed by TBLASTN searches, using the corresponding sequences from *E. coli*, *Listeria monocytogenes*, and *Staphylococcus aureus* as templates.
(0.03 MB PDF)

Table S4 Primer sequences for real-time PCR analysis.
(0.02 MB PDF)

Figure S1 γδ T cells promote monocyte survival. (*A–B*) Microscopic analysis of monocytes cultured for 18 hours under the conditions indicated, representative of three individual donors. γδ T cells in *B* were pre-labeled with PKH26 and are visualized in red; for HMB-PP treated cells three typical co-culture images are shown.
(0.32 MB PDF)

Figure S2 Monocyte-γδ T cell crosstalk depends on soluble mediators. (*A*) MFI of CD14 and CD40 for monocytes after 18 hours of culture in medium alone or with GM-CSF+IL-4, and for monocytes separated from monocyte-γδ T cell co-cultures without or with HMB-PP and anti-IFN-γ+sTNFR (data from two individual donors). (*B*) Microscopic analysis of monocytes and γδ T cells co-cultured for 18 hours in the presence of HMB-PP and the blocking reagents indicated. Data shown are representative of two individual donors.
(0.22 MB PDF)

Figure S3 Acquisition of APC markers by γδ T cell-activated monocytes depends in part on IFN-γ, TNF-α, GM-CSF, and IL-4. MFI±SEM of CD14, CD40, CD86, and HLA-DR for monocytes after 18 hours of culture under the conditions indicated (*n* = 4–8).
(0.02 MB PDF)

Figure S4 γδ T cell-activated monocytes express DC markers. (*A*) Side scatter and CD209 fluorescence for monocytes after 18 hours of culture under the conditions indicated. Results are representative of four independently assessed donors; numbers indicate the percentage of CD209^+^ monocytes. (*B*) MFI±SEM of CD83, CD206, and CD209 for monocytes after 18 hours of culture under the conditions indicated (*n* = 4–10).
(0.05 MB PDF)

Figure S5 Peritoneal γδ T cells respond to HMB-PP and promote monocytes differentiation. 250,000 peritoneal cells were cultured with HMB-PP at the indicated concentrations. (*A*) Microscopic analysis of cultures after 18 hours in the absence or presence of 100 nM HMB-PP and the blocking reagents indicated. (*B*) γδ T cell responses are shown as % of Vγ 9^+^ T cells expressing surface CD25 and CD69, and intracellular TNF-α, and as % of Vγ 9^+^ among all CD3^+^ T cells after 7 days. (*C*) Monocyte responses are shown as forward scatter; MFI of CD14, CD40, CD86, and TRAIL; and percentage of TNF-α^+^ monocytes after 18 h.
(0.37 MB PDF)
