# Supplementary material for: A Rapid Crosstalk of Human γδ T Cells and Monocytes Drives the Acute Inflammation in Bacterial Infections
Source: PLoS Pathog. 2009 Feb 20;5(2):e1000308. doi: 10.1371/journal.ppat.1000308 (PMC2637987; doi:10.1371/journal.ppat.1000308)
Supplement: Table S1 — Schematic summary of the phenotypical characterization of freshly isolated monocytes, monocytes cultured in medium or with LPS, recombinant GM-CSF+IL 4, M-CSF, or IFN-γ+TNF-α, and monocytes cultured in the presence of γδ T cells+HMB-PP, based on up to 17 independently assessed donors. Expression levels of the markers indicated were assessed after 18 hours of stimulation: −, absent or marginal; +, present; ++, strongly expressed; +++, very strongly expressed; n.d., not determined. (0.02 MB PDF) [file ppat.1000308.s001.pdf]

Table S1

	fresh monocytes	medium	LPS	GM-CSF + IL-4	M-CSF	IFN-γ + TNF-α	γδ + HMB-PP
**CD4**	+	*n.d.*	*n.d.*	*n.d.*	*n.d.*	–	–
**CD14**	++	++	++	–	+++	–	–
**CD40**	–	+	++	++	+	++	+++
**CD86**	–	+	+	+++	+	++	+++
**HLA-DR**	–	+	+++	+++	++	++	+++
**CD83**	*n.d.*	–	*n.d.*	+	*n.d.*	++	++
**CD205**		–	–	–	–	*n.d.*	–
**CD206**	–	–	–	+++	–	–	+
**CD207**	–	–	–	–	–	–	–
**CD209**	–	–	–	+++	–	–	+
**CCR2**	++	++	–	*n.d.*	*n.d.*	–	–
**CCR5**	*n.d.*	+	–	*n.d.*	*n.d.*	+	–
**CCR7**	–	–	+	*n.d.*	*n.d.*	–	–
**CXCR4**	++	++	–	*n.d.*	*n.d.*	–	–
**TNF-α**	*n.d.*	–	+++	–	–	++	+++
**TRAIL**	*n.d.*	–	++	++	+	+++	+++
**OSM**	*n.d.*	–	+++	+	–	–	++
**IFN-γ**	*n.d.*	–	–	–	–	–	–
**IL-1β**	*n.d.*	–	+++	–	–	–	–
**IL-6**	*n.d.*	–	+++	–	–	+	+
**IL-10**	*n.d.*	–	+++	–	–	–	–
**IL-12p70**	*n.d.*	–	–	*n.d.*	*n.d.*	–	–
**IL-17**	*n.d.*	–	*n.d.*	*n.d.*	*n.d.*	–	–
**IL-23**	*n.d.*	–	*n.d.*	*n.d.*	*n.d.*	–	–
**IL-27**	*n.d.*	–	–	*n.d.*	*n.d.*	–	–
**CCL2**	*n.d.*	+	+++	–	+++	++	++
**CXCL8**	*n.d.*	++	+++	+	++	++	+++
**CXCL10**	*n.d.*	–	–	–	*n.d.*	+++	+++
