# Supplementary material for: A Rapid Crosstalk of Human γδ T Cells and Monocytes Drives the Acute Inflammation in Bacterial Infections
Source: PLoS Pathog. 2009 Feb 20;5(2):e1000308. doi: 10.1371/journal.ppat.1000308 (PMC2637987; doi:10.1371/journal.ppat.1000308)
Supplement: Table S2 — Peritoneal dialysis patients with and without acute bacterial peritonitis that were analyzed in this study. n.a., not applicable. (0.02 MB PDF) [file ppat.1000308.s002.pdf]

Table S2

**no.**	**days *p.i.***	**causative agent**	**Gram**	**HMB-PP**	**material**
**1**	3, 4, 7, 14	*Bacteroides splanchnicus*	–	+	cells, fluid
**2**	1-5	coliform bacteria + *Proteus sp.*	–	+	fluid
**3**	1, 2-6	*Escherichia coli*	–	+	fluid
**4**	1-2	*Escherichia coli*	–	+	fluid
**5**	4	*Escherichia coli*	–	+	cells, fluid
**6**	5	*Escherichia coli*	–	+	cells
**7**	1-5	*Neisseria sp.*	–	+	fluid
**8**	1	*Proteus vulgaris*	–	+	fluid
**9**	1-6	*Pseudomonas sp.*	–	+	fluid
**10**	1-4	coliform bacteria + *Staphylocoocus aureus*	– / +	+	fluid
**11**	2-3	*Leclercia adecarboxylata* + *S. epidermidis*	– / +	+	cells, fluid
**12**	1-5	coryneform bacteria	+	+	fluid
**13**	1	*Staphylococcus aureus*	+	–	fluid
**14**	1-2	*Staphylococcus epidermidis*	+	–	cells
**15**	1-3	*Staphylococcus epidermidis*	+	–	cells
**16**	1	*Staphylococcus epidermidis*	+	–	cells
**17**	1	*Staphylococcus epidermidis*	+	–	cells
**18**	1	*Staphylococcus epidermidis*	+	–	cells
**19**	1	*Staphylococcus epidermidis*	+	–	cells
**20**	1	*Staphylococcus epidermidis*	+	–	fluid
**21**	1-2	*Staphylococcus epidermidis*	+	–	fluid
**22**	1-4	*Staphylococcus epidermidis*	+	–	fluid
**23**	2	*Staphylococcus epidermidis*	+	–	fluid
**24**	1-3	*Staphylococcus epidermidis*	+	–	fluid
**25**	1-4	*Staphylococcus epidermidis*	+	–	fluid
**26**	1	*Staphylococcus epidermidis*	+	–	fluid
**27**	1-2, 4	*Staphylococcus epidermidis*	+	–	fluid
**28**	1-4	*Staphylococcus epidermidis*	+	–	fluid
**29**	1-4	*Staphylococcus epidermidis*	+	–	fluid
**30**	1	*Staphylococcus epidermidis*	+	–	fluid
**31**	1-3	*Staphylococcus epidermidis*	+	–	fluid
**32**	1-3	*Staphylococcus epidermidis*	+	–	fluid
**33**	1	*Streptococcus*, α-hemolytic	+	–	cells, fluid
**34**	1	*Streptococcus*, α-hemolytic	+	–	fluid
**35**	1-4	*Streptococcus*, α-hemolytic	+	–	fluid
**36**	1-2, 6, 30	*Streptococcus*, α-hemolytic	+	–	cells
**37**	*n.a.*	non-infected, stable PD	*n.a.*	*n.a.*	cells
**38**	*n.a.*	non-infected, stable PD	*n.a.*	*n.a.*	cells
**39**	*n.a.*	non-infected, stable PD	*n.a.*	*n.a.*	cells
**40**	*n.a.*	non-infected, stable PD	*n.a.*	*n.a.*	cells, fluid
**41**	*n.a.*	non-infected, stable PD	*n.a.*	*n.a.*	fluid
**42**	*n.a.*	non-infected, stable PD	*n.a.*	*n.a.*	fluid
**43**	*n.a.*	non-infected, stable PD	*n.a.*	*n.a.*	fluid
