# Supplementary material for: A Rapid Crosstalk of Human γδ T Cells and Monocytes Drives the Acute Inflammation in Bacterial Infections
Source: PLoS Pathog. 2009 Feb 20;5(2):e1000308. doi: 10.1371/journal.ppat.1000308 (PMC2637987; doi:10.1371/journal.ppat.1000308)
Supplement: Table S3 — Distribution across bacterial genomes of coding sequences for 3-hydroxy-3-methylglutaryl-CoA (HMG-CoA) reductase (hmgr), the key enzyme of the classical mevalonate pathway; and for HMB-PP synthase (ispG) and HMB-PP reductase (ispH), two enzymes of the alternative non-mevalonate pathway of isoprenoid synthesis. Genomic and protein sequences from bacterial species of relevance for the present study were retrieved from the public servers at the National Center for Biotechnology Information (http://www.ncbi.nlm.nih.gov), Wellcome Trust Sanger Centre (http://www.sanger.ac.uk), Washington University Genome Sequencing Center (http://genome.wustl.edu), and Baylor College of Medicine Human Genome Sequencing Center (http://www.hgsc.bcm.tmc.edu). Sequence homologies were analyzed by TBLASTN searches, using the corresponding sequences from E. coli, Listeria monocytogenes, and Staphylococcus aureus as templates. (0.03 MB PDF) [file ppat.1000308.s003.pdf]

Table S3

	Gram	*hmgr*	*ispG, ispH*
**Bacteroidales: HMB-PP +**			
Alistipes putredinis	–	–	+
Bacteroides caccae	–	–	+
Bacteroides capillosus	–	–	+
Bacteroides coprocola	–	–	+
Bacteroides fragilis	–	–	+
Bacteroides intestinalis	–	–	+
Bacteroides ovatus	–	–	+
Bacteroides splanchnicus	–	?	?
Bacteroides stercoris	–	–	+
Bacteroides thetaiotaomicron	–	–	+
Bacteroides uniformis	–	–	+
Bacteroides vulgatus	–	–	+
Parabacteroides distasonis	–	–	+
Parabacteroides merdae	–	–	+
Porphyromonas gingivalis	–	–	+
Prevotella intermedia	–	–	+
Tannerella forsythensis	–	–	+
**Enterobacteriales: HMB-PP +**			
Buchnera aphidicola	–	–	+
Citrobacter koseri	–	–	+
Citrobacter rodentium	–	–	+
Dickeya dadantii	–	–	+
Enterobacter sakazakii	–	–	+
Erwinia amylovora	–	–	+
Erwinia carotovora	–	–	+
Escherichia albertii	–	–	+
Escherichia coli	–	–	+
Klebsiella oxytoca	–	–	+
Klebsiella pneumoniae	–	–	+
Leclercia adecarboxylata	–	?	?
Pantoea stewartii	–	–	+
Pectobacterium atrosepticum	–	–	+
Photorhabdus asymbiotica	–	–	+
Photorhabdus luminescens	–	–	+
Proteus mirabilis	–	–	+
Providencia stuartii	–	–	+
Salmonella bongori	–	–	+
Salmonella enterica	–	–	+
Salmonella enteritidis	–	–	+
Salmonella gallinarum	–	–	+
Salmonella paratyphi	–	–	+
Salmonella typhimurium	–	–	+
Serratia marcescens	–	–	+
Serratia proteamaculans	–	–	+
Shigella boydii	–	–	+
Shigella dysenteriae	–	–	+
Shigella flexneri	–	–	+
Shigella sonnei	–	–	+
Sodalis glossinidius	–	–	+
Wigglesworthia glossinidia	–	–	+
Yersinia enterocolitica	–	–	+
Yersinia frederiksenii	–	–	+
Yersinia intermedia	–	–	+
Yersinia mollaretii	–	–	+
Yersinia pestis	–	–	+
Yersinia pseudotuberculosis	–	–	+
Xenorhabdus boviennii	–	–	+
**Neisseriales: HMB-PP +**			
Chromobacterium violaceum	–	–	+
Neisseria gonorrhoeae	–	–	+
Neisseria lactamica	–	–	+
Neisseria meningitidis	–	–	+
**Pseudomonadales: HMB-PP +**			
Acinetobacter baumannii	–	–	+
Azotobacter vinelandii	–	–	+
Moraxella bovis	–	–	+
Pseudomonas fluorescens	–	–	+
Pseudomonas mendocina	–	–	+
Pseudomonas putida	–	–	+
Pseudomonas stutzeri	–	–	+
Pseudomonas syringae	–	–	+
Psychromonas ingrahamii	–	–	+
Psychrobacter arcticus	–	–	+
Psychrobacter cryohalolentis	–	–	+
Psychromonas ingrahamii	–	–	+

	Gram	*hmgr*	*ispG, ispH*
**Actinomycetales: HMB-PP $^{+}$**			
Acidothermus cellulolyticus	+	–	+
Actinomyces odontolyticus	+	–	+
Arthrobacter aurescens	+	–	+
Bifidobacterium adolescentis	+	–	+
Bifidobacterium longum	+	–	+
Brevibacterium linens	+	–	+
Clavibacter michiganensis	+	–	+
Collinsella aerofaciens	+	–	+
Corynebacterium diphtheriae	+	–	+
Corynebacterium efficiens	+	–	+
Corynebacterium glutamicum	+	–	+
Corynebacterium jeikeium	+	–	+
Frankia alni	+	–	+
Janibacter sp.	+	–	+
Kineococcus radiotolerans	+	–	+
Leifsonia xyli	+	–	+
Mycobacterium avium	+	–	+
Mycobacterium bovis	+	–	+
Mycobacterium leprae	+	–	+
Mycobacterium microti	+	–	+
Mycobacterium smegmatis	+	–	+
Mycobacterium tuberculosis	+	–	+
Mycobacterium ulcerans	+	–	+
Nocardia farcinica	+	–	+
Nocardioides sp.	+	–	+
Propionibacterium acnes	+	–	+
Rhodococcus equi	+	–	+
Rubrobacter xylanophilus	+	–	+
Salinispora arenicola	+	–	+
Salinispora tropica	+	–	+
Streptomyces avermitilis	+	–	+
Streptomyces coelicolor	+	–	+
Thermobifida fusca	+	–	+
Tropheryma whipplei	+	–	+
**Bacillales: HMB-PP $^{-/+}$**			
Bacillus amyloliquefaciens	+	–	+
Bacillus anthracis	+	–	+
Bacillus cereus	+	–	+
Bacillus clausii	+	–	+
Bacillus halodurans	+	–	+
Bacillus licheniformis	+	–	+
Bacillus pumilus	+	–	+
Bacillus subtilis	+	–	+
Bacillus thuringiensis	+	–	+
Bacillus weihenstephanensis	+	–	+
Exiguobacterium sibiricum	+	–	+
Geobacillus kaustophilus	+	–	+
Geobacillus thermodenitrificans	+	–	+
Listeria innocua	+	+	–
Listeria monocytogenes	+	+	+
Listeria welshimeri	+	+	–
Oceanobacillus iheyensis	+	+	–
Paenibacillus larvae	+	–	+
Staphylococcus aureus	+	+	–
Staphylococcus epidermidis	+	+	–
Staphylococcus haemolyticus	+	+	–
Staphylococcus saprophyticus	+	+	–
**Lactobacillales: HMB-PP $^{-}$**			
Enterococcus faecalis	+	+	–
Enterococcus faecium	+	+	–
Lactobacillus acidophilus	+	+	–
Lactobacillus brevis	+	+	–
Lactobacillus casei	+	+	–
Lactobacillus delbrueckii	+	+	–
Lactobacillus gasseri	+	+	–
Lactobacillus johnsonii	+	+	–
Lactobacillus plantarum	+	+	–
Lactobacillus reuteri	+	+	–
Lactobacillus sakei	+	+	–
Lactobacillus salivarius	+	+	–
Lactococcus lactis	+	+	–
Leuconostoc mesenteroides	+	+	–
Oenococcus oeni	+	+	–
Pediococcus pentosaceus	+	+	–
Streptococcus agalactiae	+	+	–
Streptococcus gordonii	+	+	–
Streptococcus mutans	+	+	–
Streptococcus pneumoniae	+	+	–
Streptococcus pyogenes	+	+	–
Streptococcus sanguinis	+	+	–
Streptococcus suis	+	+	–
Streptococcus thermophilus	+	+	–
