# Supplementary material for: A Rapid Crosstalk of Human γδ T Cells and Monocytes Drives the Acute Inflammation in Bacterial Infections
Source: PLoS Pathog. 2009 Feb 20;5(2):e1000308. doi: 10.1371/journal.ppat.1000308 (PMC2637987; doi:10.1371/journal.ppat.1000308)
Supplement: Table S4 — Primer sequences for real-time PCR analysis. (0.02 MB PDF) [file ppat.1000308.s004.pdf]

Table S4

**cyclophilin A**	
forward	5’-TCCTGGCATCTTGTCCATG-3’
reverse	5’-CCATCCAACCACTCAGTCTTG-3’
probe	5’-*FAM*-CAAATGCTGGACCCAACACAAATGG-*BHQ1*-3’
**β-actin**	
forward	5’-AGATGGCCACGGCTGCT-3’
reverse	5’-AACCGCTCATTGCCAATGG-3’
probe	5’-*FAM*-AGCTCCTCCCTGGAGAAGAGCTACGAGCT-*BHQ1*-3’
**CD40**	
forward	5’-AAGAAGCCAACCAATAAGGCC-3’
reverse	5’-CGTCGGGAAAATTGATCTCC-3’
probe	5’-*FAM*-CCCACCCCAAGCAGGAACCCC-*BHQ1*-3’
**CD86**	
forward	5’-GCGGCCTCGCAACTCTTATA-3’
reverse	5’-TCTCTTTTCTTGGTCTGTTCACTCTC-3’
probe	5’-*FAM*-ATGTGGAACCAACACAATGGAGAGGGA-*BHQ1*-3’
**HLA-DRα**	
forward	5’-GCCAACCTGGAAATCATGACA-3’
reverse	5’-AGGGCTGTTCGTGAGCACA-3’
probe	5’-*FAM*-CAACTATACTCCGATCACCAATGTACCTCCAGAG-*BHQ1*-3’
**CCR7**	
forward	5’-GCTCCAGGCACGCAACTTT-3’
reverse	5’-ACCACGACCACAGCGATGA-3’
probe	5’-*FAM*-AGCGCAACAAGGCCATCAAGGTG-*BHQ1*-3’
**TNF-α**	
forward	5’-TGGCCCAGGCAGTCAGA-3’
reverse	5’-GGTTTGCTACAACATGGGCTACA-3’
probe	5’-*FAM*-CATCTTCTCGAACCCCGAGTGACAAGC-*BHQ1*-3’
**IL-12p35**	
forward	5’-CCACTCCAGACCCAGGAATG-3’
reverse	5’-GACGGCCCTCAGCAGGT-3’
probe	5’-*FAM*-TCCCATGCCTTCACCACTCCCAA-*BHQ1*-3’
**IL-12p40**	
forward	5’-ACGGACAAGACCTCAGCCAC-3’
reverse	5’-GGGCCCGCACGCTAA-3’
probe	5’-*FAM*-TCATCTGCCGCAAAAATGCCAGC-*BHQ1*-3’
**IL-23p19**	
forward	5’-GAGCCTTCTCTGCTCCCTGAT-3’
reverse	5’-AGTTGGCTGAGGCCCAGTAG-3’
probe	5’-*FAM*-CCTGTGGGCCAGCTTCATGCCT-*BHQ1*-3’

FAM, 6-carboxyfluorescein; *BHQ1*, black hole quencher 1
