# Supplementary figures and images for: A Rapid Crosstalk of Human γδ T Cells and Monocytes Drives the Acute Inflammation in Bacterial Infections
Source: PLoS Pathog. 2009 Feb 20;5(2):e1000308. doi: 10.1371/journal.ppat.1000308 (PMC2637987; doi:10.1371/journal.ppat.1000308)

**A** $\gamma\delta$ 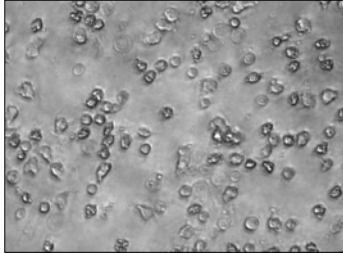 $\gamma\delta$  + HMB-PP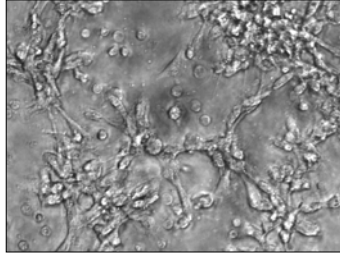

LPS

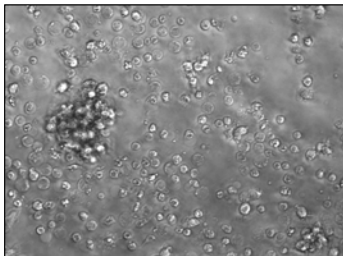

PGN

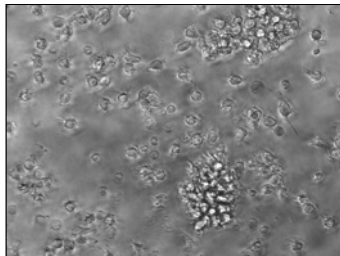

GM-CSF + IL-4

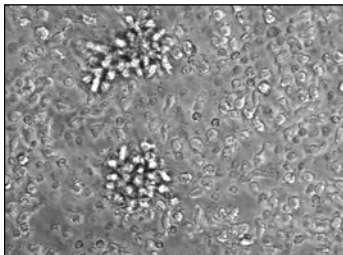

M-CSF

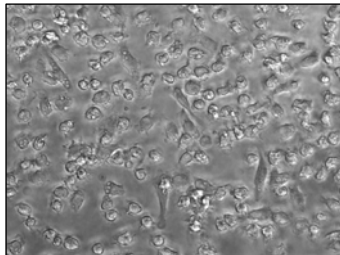**B** $\gamma\delta$ 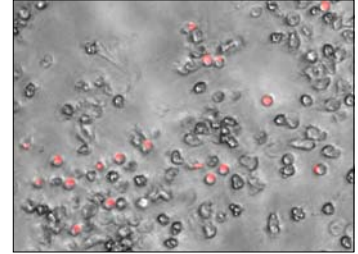 $\gamma\delta$  + HMB-PP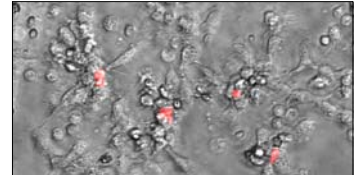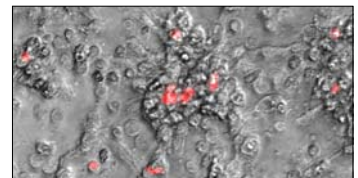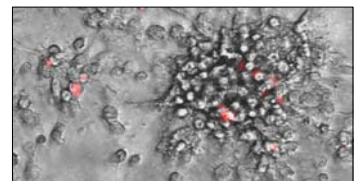

Supplement: Figure S1 — γδ T cells promote monocyte survival. (A–B) Microscopic analysis of monocytes cultured for 18 hours under the conditions indicated, representative of three individual donors. γδ T cells in B were pre-labeled with PKH26 and are visualized in red; for HMB-PP treated cells three typical co-culture images are shown. (0.32 MB PDF) [file ppat.1000308.s005.pdf]

**A**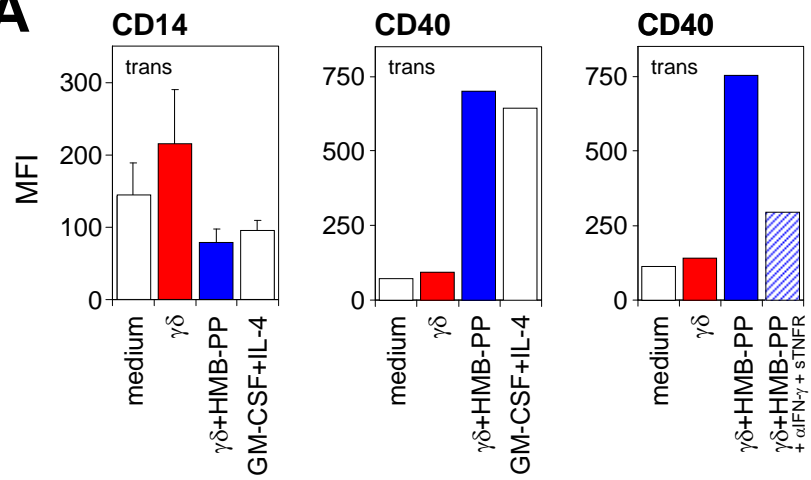**B** $\alpha$ IFN- $\gamma$ 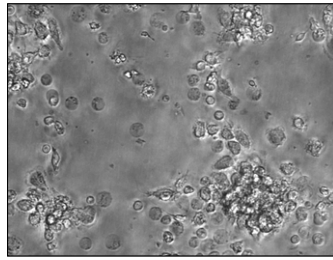

sTNFR

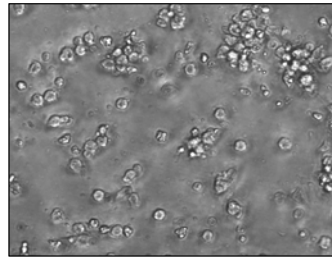 $\alpha$ IL-4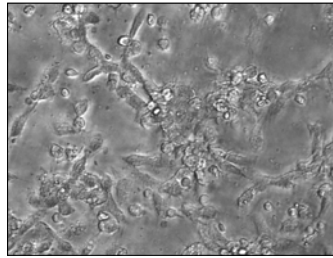 $\alpha$ GM-CSF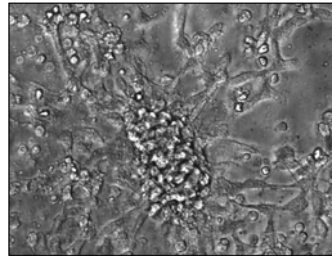

Supplement: Figure S2 — Monocyte-γδ T cell crosstalk depends on soluble mediators. (A) MFI of CD14 and CD40 for monocytes after 18 hours of culture in medium alone or with GM-CSF+IL-4, and for monocytes separated from monocyte-γδ T cell co-cultures without or with HMB-PP and anti-IFN-γ+sTNFR (data from two individual donors). (B) Microscopic analysis of monocytes and γδ T cells co-cultured for 18 hours in the presence of HMB-PP and the blocking reagents indicated. Data shown are representative of two individual donors. (0.22 MB PDF) [file ppat.1000308.s006.pdf]

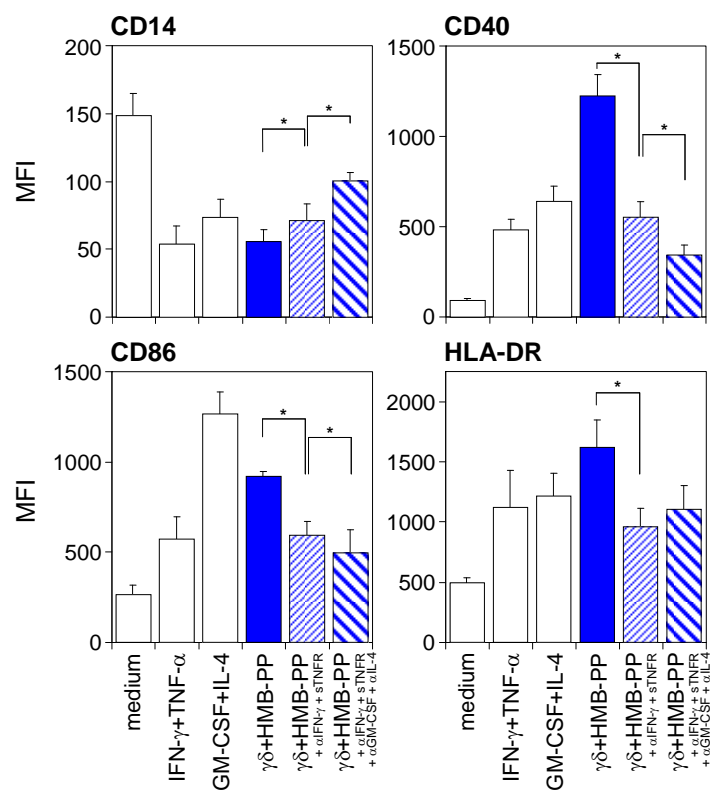

Supplement: Figure S3 — Acquisition of APC markers by γδ T cell-activated monocytes depends in part on IFN-γ, TNF-α, GM-CSF, and IL-4. MFI±SEM of CD14, CD40, CD86, and HLA-DR for monocytes after 18 hours of culture under the conditions indicated (n = 4–8). (0.02 MB PDF) [file ppat.1000308.s007.pdf]

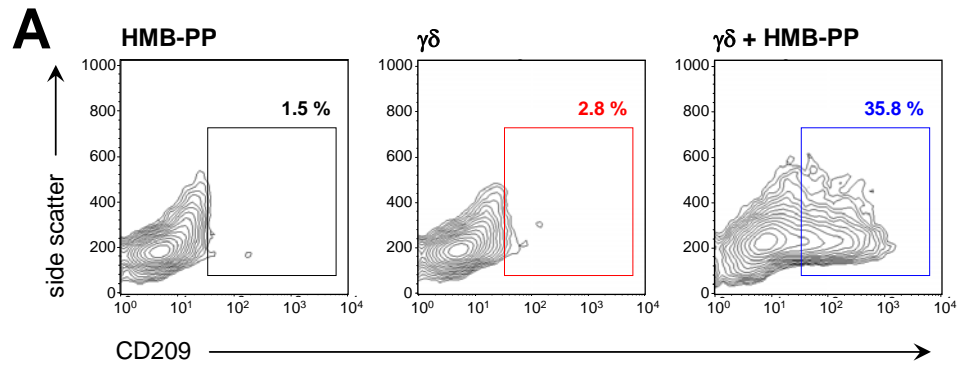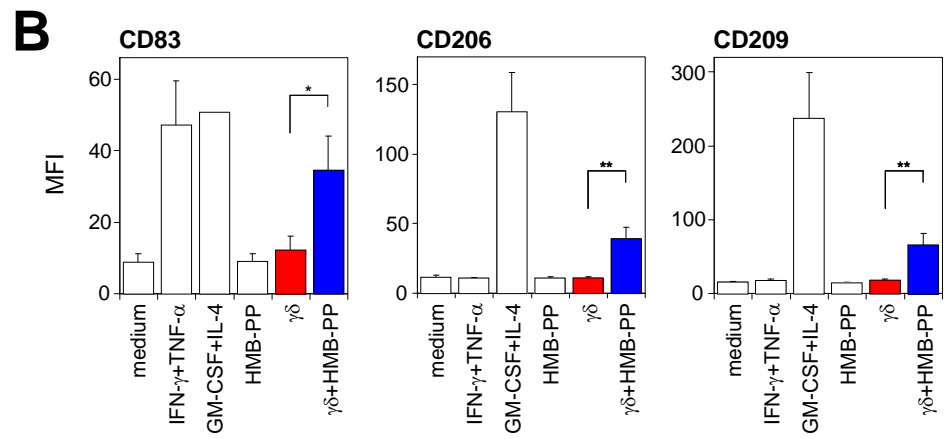

Supplement: Figure S4 — γδ T cell-activated monocytes express DC markers. (A) Side scatter and CD209 fluorescence for monocytes after 18 hours of culture under the conditions indicated. Results are representative of four independently assessed donors; numbers indicate the percentage of CD209+ monocytes. (B) MFI±SEM of CD83, CD206, and CD209 for monocytes after 18 hours of culture under the conditions indicated (n = 4–10). (0.05 MB PDF) [file ppat.1000308.s008.pdf]

**A****medium**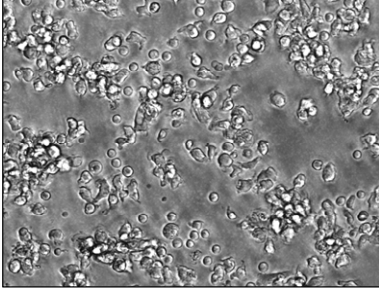**HMB-PP**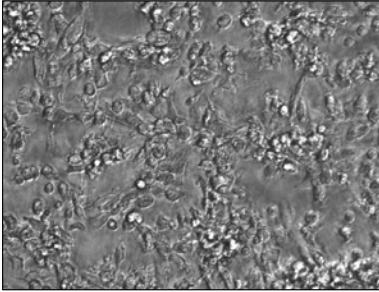**HMB-PP + anti-CD11a + anti-CD18**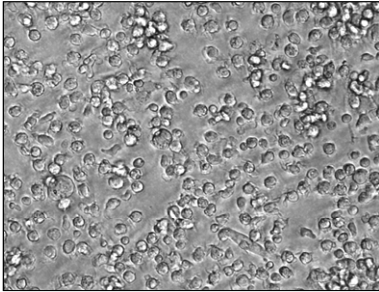**HMB-PP + anti-IFN- $\gamma$  + sTNFR**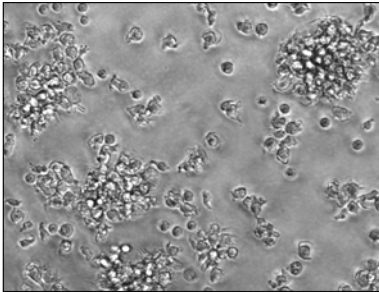**B**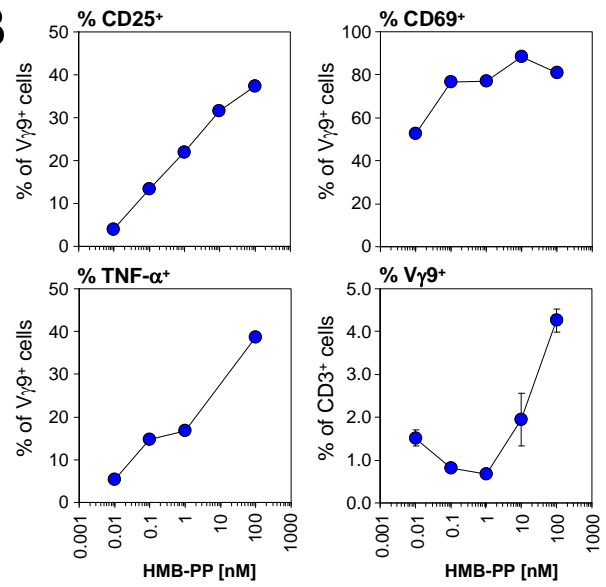**C**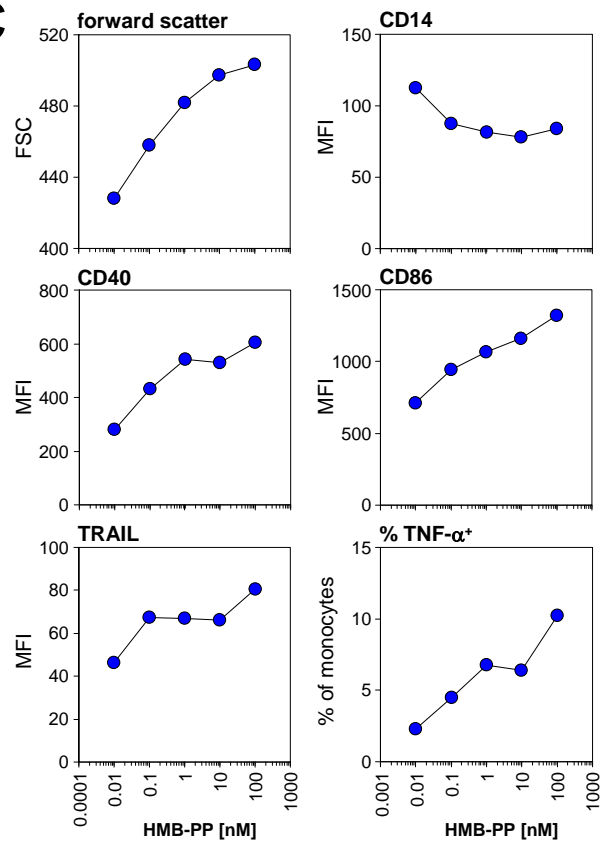

Supplement: Figure S5 — Peritoneal γδ T cells respond to HMB-PP and promote monocytes differentiation. 250,000 peritoneal cells were cultured with HMB-PP at the indicated concentrations. (A) Microscopic analysis of cultures after 18 hours in the absence or presence of 100 nM HMB-PP and the blocking reagents indicated. (B) γδ T cell responses are shown as % of Vγ 9+ T cells expressing surface CD25 and CD69, and intracellular TNF-α, and as % of Vγ 9+ among all CD3+ T cells after 7 days. (C) Monocyte responses are shown as forward scatter; MFI of CD14, CD40, CD86, and TRAIL; and percentage of TNF-α+ monocytes after 18 h. (0.37 MB PDF) [file ppat.1000308.s009.pdf]
